# Clinical Evidence Profile of Oral Chinese Patent Ethnomedicines: Protocol for a Scoping Review and Evidence Map

**DOI:** 10.2196/77741

**Published:** 2026-01-13

**Authors:** Miaomiao Li, Hui Luo, Hui Zhao, Xing Liao

**Affiliations:** 1 Medical College of Acu-Moxi and Rehabilitation, Guangzhou University of Chinese Medicine, Guangdong, China; 2 Institute of Basic Research in Clinical Medicine, China Academy of Chinese Medical Sciences, No.16, South Xiao Street, Dongzhimen Nei, Dongcheng District, Beijing, 100700, China, 86 152 0108 4286, 86 640 932 37; 3 China Tibetology Research Center, Beijing, China; 4 China Traditional Chinese Medicine Evidence-based Medical Center, China Academy of Chinese Medical Sciences, 16 South Xiao Street, Dongzhimen Nei, Dongcheng District, Beijing, China, 100700, China, 86 135 2148 3754, 86 640 932 37

**Keywords:** Chinese patent medicine, Chinese patent ethnomedicine, complementary and alternative medicine, clinical evidence, scoping review protocol, Preferred Reporting Items for Systematic Reviews and Meta-Analyses, PRISMA

## Abstract

**Background:**

Chinese patent ethnomedicines (CPEs), a form of traditional Chinese patent medicine, originate from the traditional medicines of ethnic minority groups and are widely used in clinical practice. However, existing evidence to support their application remains unclear. Therefore, to address this gap, this comprehensive scoping review will be performed to provide an overview of the available evidence on CPE preparations.

**Objective:**

This review aims to provide the evidence profile for oral CPEs. This study will elucidate the current state of the evidence with respect to these medicines and identify research gaps. The detailed steps for conducting this review are outlined in this protocol. This review will contribute to a better understanding of CPEs.

**Methods:**

This review will include clinical studies of CPEs irrespective of study design. The frameworks described by Arksey and O’Malley, Levac et al, and the Joanna Briggs Institute will be used to guide this scoping review. This review will involve six steps: (1) identifying the research question; (2) collecting information about CPEs from national related drug catalogs; (3) searching MEDLINE (via PubMed), Embase, Web of Science, Cochrane Library, and Chinese databases from inception to February 2025 to identify relevant publications; (4) screening the literature against the eligibility criteria; (5) extracting data using a predefined standardized data extraction form; and (6) summarizing, discussing, analyzing, and reporting the results. We will also present the results via data visualization techniques.

**Results:**

We will synthesize data on CPEs by conducting the scoping review, drawing the evidence maps, identifying the clinical characteristics of CPEs and how they relate to the adverse events, and highlighting the limitations and gaps in the literature. We expect to publish the results in 2027.

**Conclusions:**

The data obtained through this review could inform future research involving CPEs.

## Introduction

Chinese patent ethnomedicines (CPEs), which use indigenous medicines as raw materials, are produced based on ethnomedicine theory and the practical experience of various ethnic minority groups [1]. CPEs are a type of traditional medicine and embody the outstanding wisdom of indigenous people with respect to the use of medicinal plants, animals, and minerals. CPEs contribute significantly to the health care practices of many indigenous communities and are increasingly being used within certain communities in China [2].

China is a multiracial country with 56 nationalities and 56 different cultures, languages, and indigenous medicines due to differences in geographical and climatic conditions [3,4]; 55 of these nationalities are officially recognized as ethnic minority groups across 18 provinces of China [5]. Of China’s 55 ethnic minority groups, 47 possess either an ethnic medicine system or medical knowledge [6]. Among the indigenous medical systems, traditional Chinese medicine (TCM), also known as Han medicine, was developed by the Han people and has held an important and dominant position throughout the entire history of China’s medical development. In official documents, TCM includes both Han medicine and ethnomedicine [7]. Ethnomedicine mainly includes Tibetan traditional medicine, Mongolian traditional medicine, Uyghur traditional medicine, Miao traditional medicine, Dai traditional medicine, Yi traditional medicine, Zhuang traditional medicine, and other minority traditional medicines [8].

In recent years, the importance of ethnomedicine has increased in health care practice; in particular, the application of CPEs has become more widespread. The *Chinese Traditional Medicine Statistics*, which was published in 2015 by the National Administration of Traditional Chinese Medicine of China, and the *Investigation and Analysis of Quality Standards of Ethnomedicines in Nine Provinces of China*, which was published in 2015 by the National Medical Products Administration of China, listed 4317 CPEs produced by 161 pharmaceutical enterprises [5]. A total of 93 CPEs were included in the latest *National Reimbursement Drug List* (2021 edition). In the *Pharmacopoeia of the People’s Republic of China* (2020 edition) [9], there were 1607 kinds of Chinese patent medicines (CPMs), including 39 CPEs. These CPEs have been used to treat a range of conditions, including cardiovascular and cerebrovascular diseases, hepatobiliary system diseases, and nervous system diseases [6-9]. Furthermore, these CPEs can be administered in multiple forms, such as pills, tablets, capsules, liquids, and injections. Tibetan, Mongolian, and Uyghur traditional medicines are representative of ethnomedicines practiced among ethnic minority groups. For example, Tibetan traditional medicine is beneficial for treating digestive system disorders, rheumatic conditions, and altitude-related illnesses. Popular Tibetan patent medicines, such as the 25-flavor pearl pill (二十五味珍珠丸), are used to treat cardiovascular and cerebrovascular diseases, whereas the *ruyi zhenbao* pill (如意珍宝丸) is known to treat bone and joint diseases [10]. Mongolian patent medicines are known for their significant effects on diabetes, blood diseases, senile conditions, and concussion-induced disorders, among other diseases. For example, *wuwei shaji* powder (五味沙棘散) and *qiwei putao* powder (七味葡萄散) are used to treat chronic bronchitis and asthma in older populations. Uyghur patent medicines offer therapeutic advantages in managing skin diseases, rheumatism, and urogenital and digestive system ailments. For example, *baixuanxiatare* capsules (百癣夏塔热胶囊) are recognized for their ability to address skin diseases, including tinea handi, tinea corporis, tinea pedis, tinea versicolor, psoriasis, atopic dermatitis, herpes zoster, and acne [6].

Traditional CPMs derived from Han medicine have been well developed and widely studied because of the dominance of the Han people and their cultural cultivation and historical heritage; thus, there is an increasing amount of high-quality evidence supporting the efficacy of CPMs [11,12]. However, there is a relative lack of clinical evidence on CPEs, and there have been no systematic explorations of these medicines. To address this gap, this scoping review will identify, describe, and map the evidence profile of the application of CPEs alone or in routine biomedical practice. Previous studies have synthesized information about the statistics and analysis of CPEs listed in the *Pharmacopoeia of the People’s Republic of China* [6,9]. Building on these studies, this review aims to identify the CPEs listed in 3 national drug catalogs, systematically search for related clinical studies, and provide a visual analysis of the results to showcase the contemporary research evidence profile of CPEs. The findings of this review will highlight the available clinical research evidence on CPEs and standardized reporting frameworks, thereby informing future research efforts (Textbox 1).

Textbox 1. **Strengths and limitations of this study.**

- This review will be the first to show the clinical evidence profile of the Chinese patent ethnomedicines (CPEs) commonly used in China, including the current status of clinical research evidence, the relationship between diseases and CPEs, the clinical research collaboration network, and the clinical characteristics of the CPEs related to adverse events.
- This review will be conducted based on the framework proposed by Arksey and O’Malley [13] and Levac et al [14]. Additionally, this review will be conducted in accordance with the Joanna Briggs Institute scoping review guidelines [15]. Furthermore, this review will be reported in accordance with the PRISMA-ScR (Preferred Reporting Items for Systematic Reviews and Meta-Analyses extension for Scoping Reviews).
- The review will only include CPEs listed in the China national authoritative documents and will not include other medicines reported elsewhere.

## Methods

### Study Design and Registration

The framework described by Arksey and O’Malley [13] and Levac et al [14] and the Joanna Briggs Institute scoping review methodology will be followed and adapted during this scoping review. This review will involve six steps: (1) identifying the research question; (2) collecting information about CPEs; (3) identifying relevant studies; (4) selecting studies; (5) extracting the data; and (6) summarizing, discussing, analyzing, and reporting the results. This scoping review will be reported in accordance with the PRISMA-ScR (Preferred Reporting Items for Systematic Reviews and Meta-Analyses extension for Scoping Reviews) guidelines [16]. Additionally, this protocol was reported in accordance with the PRISMA-P (Preferred Reporting Items for Systematic Reviews and Meta-Analyses Protocols) guidelines [17]. This review protocol was registered on the Open Science Framework platform.

### Stage 1: Identifying the Research Question

This scoping review aims to provide an overview of the available evidence profile of CPEs and identify research gaps by focusing on clinical studies. Suggestions and revisions of the research question were requested from other related experimental or clinical research experts. The following questions will be addressed in our scoping review following consensus among the research team:

1. What is the current status of clinical research evidence?
2. What is the relationship between diseases and CPEs?
3. What does the clinical research collaboration network look like?
4. What are the clinical characteristics of CPEs related to adverse event (AE) features?

### Stage 2: Selecting CPEs

We selected CPEs from the *Pharmacopoeia of the People’s Republic of China* (2020 edition), *National Reimbursement Drug List* (2021 edition), and *National Essential Medicines List* (2018 edition). A total of 146 drugs were retrieved. After removing nonoral and repeated medicines, 105 CPEs remained, including Tibetan medicines (n=46, 43.8%), Mongolian medicines (n=35, 33.3%), Tibetan and Mongolian medicines (n=1, 1%), Uyghur medicines (n=18, 17.1%), Miao medicines (n=1, 1%), Yi medicines (n=1, 1%), Dai medicines (n=2, 1.9%), and Jingpo medicines (n=1, 1%). A total of 20 CPEs were included in any 2 of the aforementioned national drug catalogs. In particular, Liuwei Anxiao powder or capsules were included in the 3 national drug catalogs. Textbox 2 and Table S1 in Multimedia Appendix 1 provide basic information on the 105 CPEs included.

Textbox 2. **Information sources of the 105 included Chinese patent ethnomedicines [18-20]. Parentheses indicate that the medicine is also available in another form.**

**Tibetan medicines**

- Ershiwuwei songshi pill
- Ershiwuwei shanhu pill
- Ershiwuwei zhenzhu pill
- Shiyiwei nengxiao pill (capsule)
- Shi’erwei yishou powder
- Shisanwei bangga powder
- Shiwuwei chenxiang pill
- 70-flavor zhenzhu pill
- Qiwei tiexie pill
- Bawei chenxiang pill (capsule)
- Bawei chenxiang powder
- Jiuwei shihuihua powder
- Wuwei shexiang pill
- Renqing mangjue
- Renqing changjue
- Jiebai pill
- Cuitang pill
- Duyiwei capsule (pill)
- Changsong bawei chenxiang powder

- Dayuejing pill
- Ershiwei chenxiang pill
- Ershiwei roudoukou pill
- Ershiwuwei datang pill
- Ershiwuwei ercha pill
- Ershiwuwei lvxue pill
- Ershiwuwei zhenzhu pill
- Jiuwei niuhuang pill
- Lishukang capsule
- Liugan pill
- Liuwei nengxiao pill (capsule)
- Nuodikang pill (capsule, granule, or oral solution)
- Pazhu capsule
- Pazhu pill
- Qiwei honghuashusheng powder (pill)
- Ruyi zhenbao pill
- 35-flavor chenxiang pill
- Shanhuqishiwei pill
- Shiwei dida capsule
- Shiweiheibingpian pill
- Shiwei longdanhua granule (capsule)
- Shiwuwei heiyao pill
- Shiwuweilongdanhua pill
- Pomegranate jianwei pill (capsule)
- Pomegranate jianwei powder
- Zhituojiebai pill
- Zuozhu Daxi
- Anshen pill
- Liuwei mingmu pill
- An’erning granule
- Honghua ruyi pill

**Mongolian medicines**

- Liangxue shiwei powder (pill)
- Sanzi powder
- Sanwei tribulus powder
- Wuwei shaji powder
- Wuwei qingzhuo powder
- Liuwei muxiang powder
- Siwei tumuxiang powder
- Alatan wuwei pill
- Anshen buxin liuwei pill
- Bateri qiwei pill
- Dahuang sanwei pill
- Fengshi ershiwuwei pill
- Hanshuishi ershiyiwei powder
- Honghuaqinggan shisanwei pill
- Huangbaibawei pill
- Jixiang ankun pill
- Naru sanwei pill
- Nuangong qiwei pill (powder)
- Qinggan jiuwei pill
- Qingre bawei pill (powder and capsule)
- Roukou wuwei pill
- Surilao qingfei zhike capsule
- Diaoyuandabu ershiwuwei tang powder
- Wulan shisanweitang powder
- Xiaoji jiebai pill
- Yishen shiqiwei pill
- Xiaoer shikou powder
- Zhachong shisanwei pill
- Zhenbao pill
- Zhenzhu tongluo pill
- Shiliuwei dongqing pill
- Qiwei guangzao pill
- Qiweiputao powder
- Qingxinchenxiang bawei powder or bawei qingxinchenxiang powder
- Bawei tanxiang powder

**Tibetan and Mongolian medicines**

- Liuwei anxiao powder
- Liuwei anxiao capsule
- Aiweixin oral solution
- Baixuanxiatare pill (capsule)
- Compound gaoziban pill
- Compound muniziqi granule
- Hanchuan zupa granule
- Zukamu granule
- Hugan buzure granule
- Jianxin hemilgaozi ban’an pirated tablets
- Luobufukebiri pill

**Uyghur medicines**

- Mamulan antidiarrheal capsule
- Meiguihua oral solution
- Niaotongkakenaiqi pill
- Qingre carson granule
- Pomegranate blood-tonifying syrup
- Tongzhisurunjiang capsule (pill)
- Yanxiao dinar syrup
- Yangxindawayimixikemigao
- Yixin badiranjibuya granule
- Yajiaohadun powder

**Dai medicines**

- Dengzhan shengmai capsule

**Jingpo medicines**

- Hufengjiu

**Miao medicines**

- Yindan xinnaotong capsule

**Yi medicines**

- Lingdancao granule

### Stage 3: Identifying Relevant Studies

#### Search Strategy

We will use a 3-step systematic and comprehensive search strategy [21]. The first step will involve an initial limited search of the MEDLINE database using medicine names, including both brand and generic names, followed by an analysis of the text words contained in the titles and abstracts of the retrieved articles, as well as the index terms used to describe the articles. A second search will then be performed using the identified keywords and index terms across all the included databases. Third, the reference lists of the identified articles and reports will be searched to identify additional eligible studies. Google Scholar will be used to identify additional studies. If necessary, we will contact the authors directly to obtain further information. The search process is expected to be iterative, incorporating additional search terms and sources as the search progresses. The search strategy for the MEDLINE database is provided in Table S2 in Multimedia Appendix 1.

#### Information Source

We will systematically search PubMed, Embase (Ovid), Cochrane Library, Web of Science, China National Knowledge Infrastructure, Wanfang Data, Chongqing VIP Chinese Scientific Journal Database, and Chinese Biomedical Literature Database from inception to 2025. To ensure the feasibility and accuracy of the retrieval strategy, an information specialist at the China Academy of Chinese Medical Sciences will be consulted. The reference lists of the included articles and related systematic reviews will also be examined to identify additional eligible studies. We will include studies that evaluate eligible interventions and report predefined outcomes of interest, with no restrictions on publication language.

#### Eligibility Criteria

##### Concepts

In this scoping review, ethnomedicine is defined as the “traditional medicine” of minority communities whose knowledge and practices have been transmitted through both oral and written traditions and have evolved over the thousands of years of human existence [22,23]. This review will focus on the traditional healing practices of minority communities in China. “Biomedical” refers to modern medicine, also known as “Western medicine” or “allopathic medicine.” “Traditional and complementary medicine” is an umbrella term that is often applied to any health care practice outside the biomedical mainstream, especially in industrialized countries, including acupuncture, chiropractic, and herbal medicine [24].

##### Interventions

The eligible CPEs included in the target studies are listed in the *Pharmacopoeia of the People’s Republic of China* (2020 edition), *National Reimbursement Drug List* (2024 edition), or *National Essential Drug List* (2018 edition). We will include CPEs of any type, such as liquids, pills, gels, tablets, and extracts. We will also include studies with treatment groups combined with comparators.

##### Comparators

The eligible comparators in the target studies include placebo, no intervention, conventional Western medicine treatments, or routine care. Studies comparing different CPEs will be excluded.

##### Outcomes

The outcomes of the target studies will be categorized as follows: symptom and sign alleviation rate, physicochemical outcomes, quality of life, and other outcomes (TCM syndrome score or effective rate). We will also report safety in terms of the occurrence and types of AEs.

##### Types of Target Studies

Primary research (which involves the collection of original data for a research project), secondary research (which involves the summary or synthesis of data and literature that have been organized and published by others), and case reports on patient and physician experiences (which involve the opinions and qualitative textual data [eg, interview transcripts or open-ended responses] from patients and physicians) will be included. Other gray literature and some articles that were not published or peer reviewed will also be included if the data are complete. Short or mini reviews, narrative reviews, abstracts from conferences, letters, short communications, and comments will be excluded.

### Stage 4: Screening the Studies

**Figure 1.**
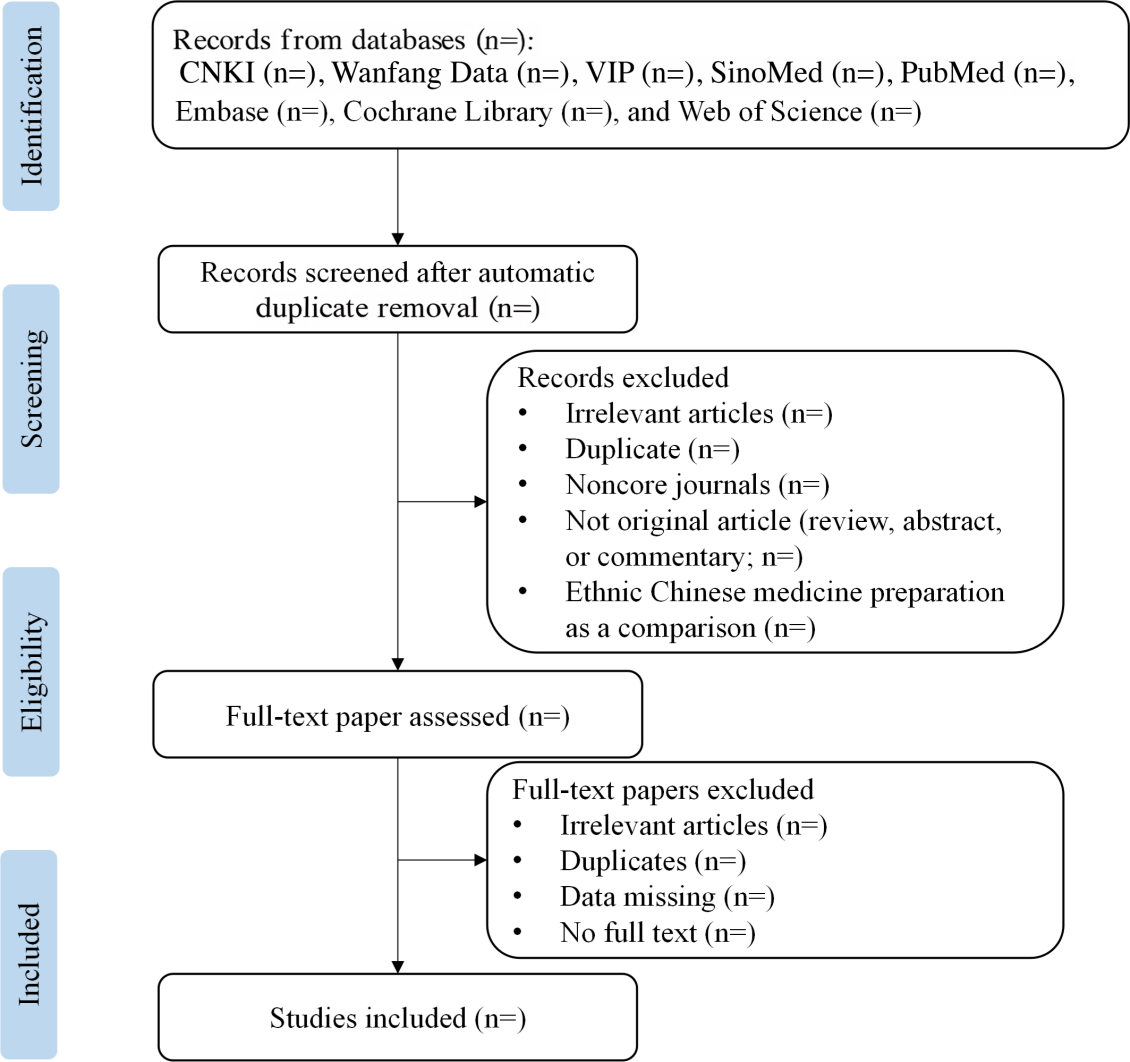
Flowchart for the selection of studies related to Chinese patent ethnomedicines. CNKI: China National Knowledge Infrastructure; SinoMed: China Biomedical Literature Service System; VIP: VIP Database for Chinese Technical Periodicals.

All identified references will be collated and uploaded into NoteExpress (version 3.4.0.8878), and duplicate records will be removed. The study selection process will be piloted independently by a reviewer using 20 studies to assess the interpretation of the eligibility criteria and the consistency of use. Following the pilot test, the eligibility criteria will be refined if necessary. During the formal process, the literature will be evaluated against the eligibility criteria; 2 trained reviewers independently and in duplicate will screen the titles and abstracts of the retrieved citations and review the full texts of studies considered potentially eligible. Disagreements will be resolved through discussion or, if necessary, adjudication from clinical experts who will be blinded to the trial results. When clarification of eligibility criteria is needed, we will contact the study authors and exclude studies by authors who do not respond. All the records that do not meet the inclusion criteria will be excluded, and the reasons for exclusion will be provided in an appendix. Multiple articles and reports from the same program will be treated as one with respect to the data extraction process and the presentation of research results. The details of the study selection process are shown in Figure 1.

### Stage 5: Charting the Data CPE

#### Data Extraction

The following data about CPEs will be extracted: market prices, formulation type, use, dosage, treatment period, and components. Additionally, treatment symptoms, signs, and diseases will be extracted into a previously developed data extraction table. The Yaozhi website will be used for data extraction of market prices, which will be performed by the first author. The senior investigator will be consulted to address any uncertainties in the data extraction process. Tables S3 and S4 in Multimedia Appendix 1 provide the detailed extraction information of the included CPEs.

#### Study Data Extraction

On the basis of the pilot-testing form, the final version of the data extraction form will be developed. The following information will be extracted from the included studies: article title, first author, publication year, sample size, participant characteristics (such as sex and age), intervention, comparator, outcomes, and study design. To facilitate the data extraction process, 2 draft charts have been prepared: one to record the characteristics of CPEs and the other to record data relevant to clinical research on CPEs, as shown in Tables S5 and S6 in Multimedia Appendix 1. The completed diagrams will be included in an appendix to the final scoping review report.

### Stage 6: Collating, Analyzing, and Reporting the Results

#### Essential Information Analysis of the Literature

Descriptive and visual analyses (eg, tables or charts, line graphs, bar charts, and bubble charts) of publication trends, CPE distribution in the literature, sample size, intervention measures, and outcomes in clinical studies will be performed. Microsoft Excel 2019 will be used to manage the data, analyze the publication trends, and examine the distribution of the CPEs. R (R Foundation for Statistical Computing) will be used to analyze outcomes. Cytoscape (Cytoscape Consortium) will be used to analyze and map the relationships between CPEs and diseases identified in the included studies. This will facilitate the analysis of which drugs are frequently used to treat specific disease combinations, thereby revealing potential treatment patterns. Quality assessment is not an integral part of scoping reviews as the aims of these reviews are to clarify concepts, identify gaps in the available evidence, and provide an overview of the literature rather than evaluate the quality of the individual studies [25].

#### Bibliometric Analysis of the Literature

The selected references will be imported into CiteSpace (version 6.3.R1) to transform the data structure. Subsequently, analyses will be performed, including coauthorship and institutional collaboration networks derived from the references, co-occurrence networks, and keyword citation bursts. The time span will encompass the period from 1994 to 2025, using a 2-year time slice and a network refinement approach that involves a pathfinder network, pruning of sliced networks, and pruning of the merged network.

#### Clinical Characteristic Identification Related to AEs

This study will apply supervised machine learning methods to identify clinical characteristics associated with AEs reported in randomized controlled trials. This will be an exploratory part of the study based on 5 to 10 published high-quality randomized controlled trials aiming to identify potential patterns rather than produce definitive predictive models. We will first prespecify the AE outcome (occurrence of any serious AE within 30 days of randomization) and harmonize variables across trials using a variable-mapping dictionary. Collected data will include baseline demographics (age and sex), disease severity indicators, treatment assignment, concomitant medications (name, dose, and timing), and trial or center identifiers. Missing data will be assessed and handled using multiple imputation performed separately within training folds to avoid information leakage. Next, through feature engineering, features related to AEs will be selected, and new features will be constructed to capture potential nonlinear relationships. A supervised learning model (random forest or logistic regression) will then be chosen, model performance will be evaluated using 10-fold cross-validation, and hyperparameters will be optimized to improve prediction accuracy. The data will be split into a 70% training set and a 30% test set; the test set will be used to validate the model’s generalization ability and analyze feature importance to identify the clinical features that contribute most to the prediction of AEs.

Before analysis, all trial datasets will undergo a standardized data quality control. We will compile a detailed data dictionary documenting variable names, units, coding schemes, and provenance (original case report form field, derived variable definition, and trial identifier). The quality assessment steps will include range and logic checks, detection and resolution of duplicate records, verification of date and temporal consistency (eg, treatment start and stop dates vs AE onset), and cross-checking of AE coding against source documents where available. The variables with excessive missingness (prespecified threshold, eg, >50%) will be considered for exclusion or treated in sensitivity analyses. All data cleaning steps and data transformations will be logged in an audit trail to ensure reproducibility. Regarding data availability, we will respect original trial consent and data sharing agreements: only deidentified datasets approved for secondary analysis will be included.

## Results

We will synthesize data on CPEs by conducting the scoping review, drawing the evidence maps, identifying the clinical characteristics related to AEs, and highlighting the limitations and gaps in the literature. CPE selection was completed in 2024. Data collection for clinical studies is expected to begin in December 2025 and finish in December 2026. As of December 2025, data analysis has not yet started, and results are expected to be published in December 2027.

## Discussion

### Anticipated Findings

Current ethnomedicine studies have focused on identifying classic, well-known prescriptions in ancient indigenous medicine texts and conducting textual research on them through the lens of classic literature rather than clinical research [26,27]. Therefore, research that focuses on analyzing the clinical characteristics of CPEs should be considered. To our knowledge, this scoping review is the first to systematically identify indigenous medicines endorsed by national authoritative drug catalogs and provide an overview of contemporary clinical research evidence. We will analyze the characteristics of previous clinical studies; explore the relationship between indigenous medicines and disorders based on contemporary research; and, ultimately, provide valuable insights and inspiration for future studies concerning CPEs.

### The Current Status of CPEs

As an integral aspect of TCM, ethnomedicine continues to be highly valuable in health care and has emerged as a complementary system to contemporary medical practices. Over time, through enduring application and refinement, it has led to the development of a variety of unique CPEs, which have played important roles in the prevention and treatment of chronic conditions, challenging illnesses, and endemic diseases in regions inhabited by ethnic minority groups. Many ethnic minority groups rely on CPEs as an adjuvant or alternative to conventional medical treatments.

However, ethnic medicine is not a mainstream medicine similar to Han Chinese medicine. The minority language, obscure medical theory system, unique processing methods, and wide regional differences have become barriers to the promotion and application of ethnic medicine. In particular, many CPEs contain toxic ingredients, such as *zuotai* (佐太), nux vomica (马钱子), aconite (乌头), aristolochic acid (马兜铃酸), and iron powder (铁粉). Among these, *zuotai* (佐太) is a preparation formed by the special processing of mercury by Tibetans. As an active ingredient, it has been used to treat cardiovascular disorders, liver disorders, and gastrointestinal disorders combined with Tibetan herbal medicine for 1300 years [28,29]. However, relevant studies have shown that *zuotai* can cause damage to the liver and kidney tissues of rats, especially long-term toxic effects on the spleen [30], which means that a greater number of medicines containing *zuotai* cannot be accepted and applied by a wider range of clinicians outside of minority groups. The same is true for other medicines containing toxic ingredients. Therefore, based on a scoping review of the existing literature, this study will conduct an in-depth analysis of the clinical characteristics of AEs using the collected clinical research evidence base. By identifying key clinical features associated with AEs, this study aims to enhance the safety profile and optimize the clinical treatment outcomes of CPEs.

At present, the exact mechanisms underlying the effects of most CPEs and their disease prevention effects have yet to be fully elucidated. Nonetheless, public enthusiasm for these treatments persists [8]. The natural source of these drugs makes them readily acceptable to the general population [31]. However, few clinical studies have examined the use of CPEs, and the existing evidence supporting their efficacy is inconclusive. Moreover, highly efficient and safe prescriptions have not been thoroughly explored, disseminated, or used. The knowledge regarding the use of ethnomedicine in clinical use and research of CPEs within modern medical practice is also unclear among most medical professionals [32,33]. These factors make it difficult to assess and recognize the potential value of CPEs or explore new avenues to enhance clinical practice. Consequently, there is an imperative need for a comprehensive review and analysis of relevant clinical studies. This effort is vital not only to protect and pass down the knowledge of traditional CPE use but also to provide guidance for ongoing research and development in the field of ethnomedicine.

### Implications for Future Research

In the future, we will conduct targeted analyses (eg, systematic reviews or network meta-analyses) to scientifically evaluate the efficacy and safety of CPEs. Additionally, we will perform comparative analyses between similar medicinal products to determine the specific therapeutic advantages of CPEs. To further highlight the characteristics and advantages of CPEs and the role of ethnic Chinese medicine in the health care field, the following suggestions are proposed. The first is to cultivate researchers with the cross-subject background of TCM, ethnic medicine, and Chinese medical literature, among other related fields, to find the intersection of the 2 theories (TCM and Chinese ethnomedicine) from the perspective of historical traceability and interpret and transform the theory of ethnic medicine into the concept understood in Han Chinese medicine to deeply master the theory of ethnic medicine and promote the complementarity of the discipline. The second suggestion is to select the priority disorders that classic CPEs treat, identify the disease stage in which these therapies demonstrate comparative therapeutic advantages, and precisely establish complementary advantages in conjunction with Han Chinese medicine. Third, given that ethnic medicines contain many toxic components, with the patient’s informed consent and full consideration of their wishes, pilot studies of advantageous ethnic medicines can be conducted for end-stage patients with critical diseases such as cancer or rare diseases that lack effective therapy in modern medicine. The fourth suggestion is to promote the localization of ethnic medicine production; compare the processing technology of ethnic drugs with that of modern processing methods; carry out the interdisciplinary integration of research methodologies across different medical fields; and, therefore, further understand the value of the processing method of ethnic medicines to increase their acceptability and dissemination.

### Strengths and Limitations

Developing a protocol before conducting a scoping review is beneficial for several reasons [17]. First, protocols help predict potential challenges that may arise during the review. Second, protocols prevent arbitrary decisions related to the inclusion criteria and the data extraction process. Third, protocols mitigate the risk of selective reporting. Fourth, protocols minimize the likelihood of study duplication. For this study, we preliminarily identified some indigenous medicines and will retrieve relevant literature, screen the studies, and extract the data.

This protocol describes an exploratory research framework for secondary literature regarding CPEs in which the evidence will be evaluated via comparative analysis rather than quantitative synthesis. Furthermore, this review will only provide a clinical overview of the CPEs listed in Chinese national authoritative documents and will not include other medicines reported elsewhere.

### Conclusions

This paper presents a comprehensive scoping review protocol aimed at evaluating the clinical evidence profile of oral CPEs. The review will systematically identify, describe, and map the evidence related to the application of CPEs in clinical practice with a focus on addressing the current lack of clear evidence and identifying research gaps. The findings will contribute to a better understanding of CPEs and provide valuable insights for future research and development in the field of ethnomedicine.

## Supplementary material

10.2196/77741 Multimedia Appendix 1 Basic information, search strategy, market prices, composition details, and characteristics tables of the 105 traditional Chinese ethnic patent medicines.

## Abbreviations

AE: adverse event
CPE: Chinese patent ethnomedicine
CPM: Chinese patent medicine
PRISMA-P: Preferred Reporting Items for Systematic Reviews and Meta-Analyses Protocols
PRISMA-ScR: Preferred Reporting Items for Systematic Reviews and Meta-Analyses extension for Scoping Reviews
TCM: traditional Chinese medicine

## References

[1] Zhu Z, Wang T, Fu D, Gui Y, Wang J, Cui T (2016). Innovative development path of ethnomedicines: an overview of ethnomedicines in China. Front Med.

[2] Li J, Zhang Y (2021). Inheritance and innovation of ethnic medicine under the vision of the Chinese national community. World J Tradit Chin Med.

[3] Chen R, Duan ZY, Duan XH, Chen QH, Zheng J (2022). Progress in research on gut microbiota in ethnic minorities in China and consideration of intervention strategies based on ethnic medicine: a review. Front Cell Infect Microbiol.

[4] Wanzala W, Minyoso SI (2024). Ethnomedicines in the 21st century: challenges and opportunities in the contemporary world. J Med Herbs Ethnomed.

[5] Li Z, Li C, Zhang X (2020). Policies and problems of modernizing ethnomedicine in China: a focus on the Yi and Dai traditional medicines of Yunnan Province. Evid Based Complement Alternat Med.

[6] Zhao W, Kong L, Wang X, Liu Q, Wang Y, Wang J (2025). Efficacy and safety of Chinese patent medicines for allergic rhinitis based on 2020 Chinese Pharmacopoeia: a protocol for systematic review and meta-analysis of randomized controlled trials. Syst Rev.

[7] (2018). Transcript of the press conference on “several opinions on strengthening the work of ethnic minority medicine in the new era”. National Administration of Traditional Chinese Medicine.

[8] Pan SY, Litscher G, Gao SH (2014). Historical perspective of traditional indigenous medical practices: the current renaissance and conservation of herbal resources. Evid Based Complement Alternat Med.

[9] Yin XB, Qu CH, Dong XX, Shen MR, Ni J (2022). Preparation regularity of Chinese patent medicine in Chinese Pharmacopoeia (2020 edition, Vol.Ⅰ). Zhongguo Zhong Yao Za Zhi.

[10] Lu S, Han F, Kong XW, Fan X (2017). Analysis of the reasonability of clinical use of folk medicine in the Third Affiliated Hospital of Beijing University of Chinese Medicine. Clin Med J.

[11] Liu S, Yao C, Xie J (2023). Effect of an herbal-based injection on 28-day mortality in patients with sepsis: the EXIT-SEP randomized clinical trial. JAMA Intern Med.

[12] Zhang M, Liu Y, Xu M (2019). Carotid artery plaque intervention with Tongxinluo capsule (CAPITAL): a multicenter randomized double-blind parallel-group placebo-controlled study. Sci Rep.

[13] Arksey H, O’Malley L (2005). Scoping studies: towards a methodological framework. Int J Soc Res Methodol.

[14] Levac D, Colquhoun H, O’Brien KK (2010). Scoping studies: advancing the methodology. Implement Sci.

[15] Peters MD, Godfrey C, McInerney P, Munn Z, Tricco AC, Khalil H, Aromataris E, Munn Z (2020). JBI Manual for Evidence Synthesis.

[16] Tricco AC, Lillie E, Zarin W (2018). PRISMA extension for scoping reviews (PRISMA-ScR): checklist and explanation. Ann Intern Med.

[17] Moher D, Shamseer L, Clarke M (2015). Preferred reporting items for systematic review and meta-analysis protocols (PRISMA-P) 2015 statement. Syst Rev.

[18] National Pharmacopoeia Commission (2020). Pharmacopoeia of the People’s Republic of China.

[19] National Healthcare Security Administration. National Reimbursement Drug List.

[20] (2018). National essential drug directory. National Health Commission of the People’s Republic of China.

[21] Blignault I, Hunter J, Mumford J (2018). Integration of Indigenous healing practices with western biomedicine in Australia, Canada, New Zealand and the United States of America: a scoping review protocol. JBI Database System Rev Implement Rep.

[22] Chattopadhyay D (2009). Ethnomedicinal phytophores in disease management. Internat J Biomed Pharmaceut Sci.

[23] Chattopadhyay D (2010). Ethnomedicine: A Source of Complementary Therapeutics.

[24] (2013). WHO traditional medicine strategy: 2014-2023. https://www.who.int/publications/i/item/9789241506096.

[25] Munn Z, Peters MD, Stern C, Tufanaru C, McArthur A, Aromataris E (2018). Systematic review or scoping review? Guidance for authors when choosing between a systematic or scoping review approach. BMC Med Res Methodol.

[26] Ding K, Cheng L, Zha B, Bao WE, Zhang LJ, Xie YM (2021). Methods and key points of literature collation and mining of classic prescriptions in ethnic medicine. Zhongguo Zhong Yao Za Zhi.

[27] Sun CQ, Li YY, Xie ZN, Xie YM (2021). General screening principles and inheritance paths of classic prescriptions in Tibetan medicine. Zhongguo Zhong Yao Za Zhi.

[28] Kannan N, Nv AK, Balaji S (2025). Chemical, elemental, morphological and toxicological characteristics of traditional Indian Siddha formulation: Kasthuri Karuppu. Adv Tradit Med.

[29] Dou GG, Wei L, Zhou W, Zhang Y (2024). Process development and research status of Tibetan medicine zuotai (mercury preparation). World Sci Technol-Mod Tradit Chin Med.

[30] Qiao Y, Chen H, Guo J (2024). A study on the effects of metacinnabar (β-HgS) on weight and appetite recovery in stressed mice. J Ethnopharmacol.

[31] Gupta AA, Jadhav A, Bhola N, Agrawal P (2022). Insight of ethnomedicines in dentistry: a brief review. Cureus.

[32] Zhu Z, Fu D, Gui Y (2017). Innovative development path of ethnomedicines: the interpretation of the path. Front Med.

[33] Ping N (2019). Study on the development status and protection countermeasures of naxi ethnic medicine. https://link.oversea.cnki.net/doi/10.27200/d.cnki.gkmlu.2019.001264.

