# Supplementary material for: Clinical Evidence Profile of Oral Chinese Patent Ethnomedicines: Protocol for a Scoping Review and Evidence Map
Source: JMIR Res Protoc. 2026 Jan 13;15:e77741. doi: 10.2196/77741 (PMC12810741; doi:10.2196/77741)
Supplement: Multimedia Appendix 1 [file resprot-v15-e77741-s001.docx]

**Table S1**

The Basic Information of 105 CPEs.

**Table S2**

Proposed search strategy to search MEDLINE (PubMed).

**Table S3**

The Market prices of CPE.

**Table S4**

The composition of 105 CPEs.

**Table S5**

Characteristics table of Chinese patent ethnomedicines.

**Table S6**

Clinical research characteristics table of CPEs.

**Table S1**

Basic information of 105 Traditional Chinese ethnic patent medicines

| Traditional Chinese ethnic patent medicines | The main efficay of the drugs | The main symptoms and disease of drug treatment |
| --- | --- | --- |
| Ershiwuwei Songshi Wan | Clearing heat and detoxifying, soothing liver and gallbladder, removing blood stasis | Liver depression, qi stagnation, blood stasis, liver poisoning, liver pain, liver cirrhosis, liver seepage, various acute and chronic hepatitis and cholecystitis |
| Ershiwuwei Shanhu Wan | Open orifices, dredge collaterals and relieve pain | Neurological disorders, unconsciousness, numbness, dizziness, brain pain, irregular blood pressure, headache, epilepsy and various neuropathic pain |
| Shiyiwei Nengxiao Wan（Capsule） | Remove blood stasis, promote blood circulation, stimulate the menstrual flow and expedite child delivery | For amenorrhea, irregular menstruation, dystocia, abnormal placenta, postpartum blood stasis and abdominal pain |
| Shi’erwei Yishou Powder | Clearing heat and detoxification, epidemic prevention | Plague, influenza, Japanese encephalitis, dysentery, calentura and fever |
| Shisanwei Bangga Powder | Clearing heat and detoxification, cooling liver and benefiting gallbladder | Hot "Chiba" disease, cholecystitis, icteric hepatitis |
| Shiwuwei Chenxiang Wan | Regulating qi and blood, relieving cough and calming nerves | Stagnation of Qi and blood, chest pain, dry cough, shortness of breath, insomnia |
| Seventy-flavor Zhenzhu Wan | Soothe the mind, calm, dredge meridians and activate collaterals, harmonize qi and blood, restoring consciousness and open the orifice | For cardiovascular and cerebrovascular system and nervous system diseases, irregular menstruation and blood circulation; Stroke, paralysis, hemiplegia, epilepsy, cerebral hemorrhage, concussion, heart disease, hypertension and neurological disorders |
| Qiwei Tiexie Wan | promote the circulation of qi and invigorate the circulation of blood, calm the liver, clear away heat and relieve pain | For liver pain and intumescentia |
| Bawei Chenxiang Powder(Pill、Capsule、Wan） | Clearing away the heart-fire, nourishing heart, calming mind and opening orifice | It is used for heat attack, delirium, coronary heart disease and angina pectoris |
| Jiuwei Shihuihua Powder | Clearing heat, detoxifying, relieving cough and calming nerves | For children with pneumonia, high fever, irritability and cough |
| Wuwei Shexiang Wan | Antiinflammatory,analgesic, dispelling wind | For tonsillitis, angina, influenza, anthrax, rheumatoid arthritis, neuralgia, stomachache, toothache. |
| Renqing Mangjue | Clearing heat and detoxification, benefiting liver and stomach, brightening eyes and waking up spirit, healing sores, nourishing and strengthening the body | It is used for various poisoning diseases such as natural poison, food poison and preparation poison, gastrointestinal ulcer, acute and chronic gastroenteritis, atrophic gastritis, ascites, leprosy, etc |
| Renqing Changjue | Clearing away heat and toxic material ，nourishing and tonifying | Obsolescence gastroenteritis, ulcer, atrophic gastritis, various poisoning, syphilis, leprosy, obsolescence fever, anthrax, furuncle pain, dry yellow water, suppuration, etc |
| Liuwei Anxiao Powder（Tibetan/Mongolian medicine） | Harmonizing the stomach, tonifying the spleen, eliminating accumulation and guiding stagnation, invigorating the circulation of blood and relieving pain | It is used for epigastric fullness, dyspepsia, constipation and dysmenorrhea caused by disharmony between spleen and stomach,and internal stagnation |
| Jiebai Wan | Strengthen the spleen and stomach, relieve pain, stop vomiting and distinguish turbid secretion | It is used for chest and abdomen distension, epigastric pain, indigestion, nausea, vomiting, diarrhea and adverse urination |
| Cuitang Wan | Clearing heat and relieving exterior symptoms, relieving cough and pain | For the beginning of a cold, cough, headache, arthralgia, and prevention of influenza. |
| Changsong Bawei Chenxiang Powder | Clearing heart and calming mind, promoting the circulation of qi and reducing blood pressure | For irregular Qi and blood, chest tightness, shortness of breath, chest and back pain, hypertension and cardiovascular diseases |
| Dayuejing Wan | Clearing away heat and toxic material,helping digestion | It is used for poisoning, gastrointestinal ulcer, hematemesis or hematochezia, to remove hidden heat, old heat, fluctuating heat, dyspepsia, acute abdominal pain, insect disease, yellow water disease, ruffian tumor and other complications |
| Ershiwei Chenxiang Wan | Harmonizing Qi and blood, calming mind and nerves | It is used for hemiplegia, hypertension, mental disorder, crooked mouth and eyes, limb numbness and insomnia |
| Ershiwei Roudoukou Wan | calming mind and nerves | It is used for nervous system disease, mental disorder, agitated, trance, insomnia, dizziness, forgetfulness, tinnitus, tremor and palpitation |
| Ershiwuwei Datang Wan | Mixing dragon, Chiba, bacon and appetizer in harmonious proportion, stimulating the appetite | It is used for body weight of aeipathia, loss of appetite and excessive menstruation |
| Ershiwuwei ErCha Wan | Dispel wind and arthralgia, diminish inflammation and relieve pain, dry yellow water | For nervous system diseases, arthrolithiasis, rheumatoid arthritis, joint swelling, pain and deformation, limb stiffness, yellow water disease, lower extremity vasculitis, etc |
| Ershiwuwei Lvxue Wan | Dispelling wind, dehumidification, dry yellow water | It is used for swelling, pain, deformation and yellow water accumulation of limb joints caused by arthritis, rheumatoid arthritis, arthrolithiasis and arthralgia |
| Ershiwuwei zhenzhu Wan | Calming the mind and opening orifice | It is used for stroke, hemiplegia, crooked mouth and eyes, unconsciousness, mental disorder, delirium, madness, etc |
| Jiuwei Niuhuang Wan | Removing heat from the liver | For large liver, liver pain, nausea and red eyes. Various hepatitis, allergic diseases, digestive system diseases |
| Lishukang Capsule | Invigorating the spleen and tonifying the kidney, generating essence and nourishing blood, benefiting the lung and calming the heart | It is used for dizziness, palpitation, shortness of breath, frequent wheezing, poor appetite, weak waist and knees, easy fatigue, high altitude reaction and high altitude polycythemia |
| LiuGanWan | Clearing away heat and toxic material | For influenza, runny nose, headache and cough, body aches, inflammation and fever. |
| LiuWei Nengxiao Wan（Capsule） | Help digestion, reducing the swelling, regulating wind and harmonizing stomach | It is used for food poisoning, indigestion of accumulated food, stomach pain, chest and abdomen swelling, dry stool, dystocia, difficult shedding of secundinat, etc |
| Nuodikang Pill（Capsule、Granule、Oral solution） | Supplementing qi and activating blood circulation, dredging pulse and relieving pain | It is used for chest arthralgia, manifested as chest tightness, tingling or dull pain, palpitation, shortness of breath, mental fatigue, lack of breath, lazy speech, dizziness, etc. Coronary heart disease and angina pectoris are also effective. |
| Pazhu Wan(Capsule） | Invigorating stomach and dispersing cold, removing phlegm, breaking pimples and tumors, nourishing prosperity and strength | It is used for xiphoid phlegm disease, dyspepsia caused by gastric ruff tumor, gastric distention, gastric burning, pantothenic acid, stomach and liver discomfort. |
| Qiwei Honghuashusheng Powder（Pill） | Clearing heat and diminishing inflammation, protecting liver and eliminating jaundice | It is used for old and new liver diseases, increased liver blood formed via internal lesion caused by overexertion , hepatomegaly, yellow sclera and V loss of appetite caused by fatigue |
| Ruyi Zhenbao Wan | Clear away heat, awaken the brain and open the orifices, relax tendons and collaterals, and dry yellow water | It is used for plague fever, old fever, nervous system diseases, limb numbness, paralysis, skew mouth and eyes, unconsciousness, arthralgia, arthrolithiasis, limb rigidity and unfavorable joints. It has good effect on nervous system diseases. |
| Thirty five flavor Chenxiang Wan | Cleaning distemper, dispelling the wind ，benefiting the lung, clearing away arthralgia | It is used for the initial onset of fever, chronic lung disease, lung iron cloth disease, cough, Qi inversion, arthralgia, Qi and blood congestion, etc |
| ShanHuQiShiWei Wan | Calming the mind and nerves, relieving the fears and regulating your blood | For cerebral thrombosis, cerebral hemorrhage, coronary heart disease, limb paralysis, tachycardia or bradycardia, hypertension, poliomyelitis, epilepsy and various neuritis. It has special effects on brain, nerve and heart diseases |
| Shiwei Dida Capsule | Soothing the liver and regulating qi, clearing away heat and poison, promoting gallbladder and dissolving stones | For hypochondriac pain caused by damp heat of liver and gallbladder, symptoms include dull pain or colic in the right upper abdomen, bitter mouth, nausea, belching, pantothenic acid and abdominal distention; Chronic cholecystitis or cholelithiasis with the above symptoms |
| Shiweiheibingpian Wan | Warming the stomach and eliminating food, breaking the accumulation and benefiting the gallbladder | For indigestion, nausea, bacon tumor, cholecystitis, gallstones, cold gall disease and jaundice |
| Shiwei Longdanhua Granule（Capsule） | Clearing heat and resolving phlegm, relieving cough and asthma | It is used for cough, wheezing, yellow phlegm or fever, runny nose, sore throat, thirst, yellow urine, dry stool caused by phlegm heat blocking the lung. Acute bronchitis,acute attack  of chronic bronchitis |
| Shiwuwei Heiyao Wan | Dissipate cold and dissipate food, break blood stasis and dissipate accumulation | For chronic gastroenteritis, gastric bleeding, stomach cold pain, dyspepsia, loss of appetite, vomiting and diarrhea, abdominal mass and belching frequently |
| ShiWuWeiLongDanHua Wan | Clearing heat and regulating lung, relieving cough and resolving phlegm | It is used for cough, asthma and hoarseness caused by bronchitis |
| Pomegranate Jianwei Wan(Pill、Capsule、Powder） | Warming stomach and benefiting fire | For indigestion, loss of appetite, fear for chills, diarrhea, etc |
| ZhituoJiebai Wan | Clearing stomach heat, resisting stomach acid and relieving cough | For chronic gastritis, stomach pain, vomiting sour water, cough, hoarseness, cold in stomach and poor breathing |
| Zuozhu Daxi | Soothing the liver, strengthening the stomach, clearing away heat, healing ulcers and reducing swelling | It is used for epigastric noise, burning pain, liver heat pain, dyspepsia, hiccup, vomiting and diarrhea of bile, bad blood and smoke juice, acute abdominal pain, yellow water disease, visceral tumors, food poisoning, old medical diseases, edema, oedema, etc |
| Anshen Wan | Nourishing the mind, calming the mind and restraining the wind | For neurosis, delirium, dreaminess, tinnitus, palpitation, trembling, madness, dumb knot |
| Liuwei Mingmu Wan | Clearing heat and purging fire, suppressing liver and brightening eyes | Used for redness, swelling, pain, fear of light, tears and unclear vision caused by inflammation of liver-fire |
| Shiliuwei Dongqing Wan | Relaxing the chest and soothing Qi, stoping cough and asthma | For chest fullness, abdominal distension, dizziness, edema, cold cough, asthma due to excessive phlegm |
| Qiwei Guangzao Wan | Nourishing the heart, replenishing qi and calming the mind | For chest tightness, pain, palpitation, shortness of breath, restlessness of mind, insomnia and forgetfulness |
| Qiweiputao Powder | Clearing away the lung-heat，stopping cough and asthma | It is used for cough due to consumptive disease, asthma due to ole age and fullness and tightness of chest |
| Qingxinchenxiang Bawei Powder/Bawei Qingxinchenxiang Powder | Clearing heart and lungs, smoothing Qi, easing mood and calming nerves | Used for heart and lung fire, chest tightness, chest and flank pain, palpitation and shortness of breath |
| Bawei Tanxiang Powder | Clearing heat and moistening lung, relieving cough and resolving phlegm | It is used for cough with lung heat and pus in sputum |
| Sanzisan | Clearing heat, cooling blood and detoxifying | For febrile diseases, blood-heat, new or long fever |
| Sanwei tribulus Powder | Clearing damp heat and diuresis | It is used for hot and humid in bottom pour, hot and painful urination |
| Wuwei Shaji Powder | Clearing heat and removing phlegm, relieving cough and asthma | It is used for long-term cough of lung heat, asthma and phlegm, fullness in the chest, chest and flank pain, and chronic bronchitis with the above symptoms |
| WuweiQingzhuo Powder | Opening stagnation, eliminating food and warming the stomach | For loss of appetite, indigestion, fullness and cold pain for gastral cavity,belching, abdominal distention and diarrhea |
| Liuwei Muxiang Powder | Opening stagnation, promoting the circulation of Qi and relieving pain | It is used for stomachache fullness pain, acid swallowing, belching, abdominal distention, abdominal pain and obstructed stool caused by mixed cold and heat, qi depression of middle-jiao |
| Siwei Tumuxiang Powder | Clear away the plague and relieve the symptoms on the body surface | It is used in the early stage of plague, chills and fever, headache and cough, sore throat, chest and flank pain |
| Alatan Wuwei Wan | Strengthening the stomach and promoting digestion | It is used for hot gastrointestinal tract, indigestion of overnight food, hepatobiliary fever and jaundice |
| Anshen Buxin Liuwei Wan | Calming the mind | It is used for palpitation and shortness of breath |
| Bateri Qiwei Wan | Removing plague and detoxify, eliminating "viscosity", relieving pain, dispersing blood stasis and stoping dysentery | It is used for epidemic fever, encephalitis, red and white dysentery, diphtheria, yellow eyes, hoarseness and twitch |
| Dahuang Sanwei Pill | Clearing away heat and loosing the bowels | For heat stagnation, constipation, stomach distention, stomach pain, nausea and acid swallowing |
| FengShi Ershiwuwei Wan | Eliminating dampness and dispersing blood stasis | For wandering pain, arthritis, rheumatoid |
| Hanshuishi Ershiyiwei Powder | Dispelling Baori" disease | For "Baori" disease, belching, acid swallowing, chest and back pain, qi stagnation and blood stasis, blood heat trapping stomach in the early and middle stages |
| Honghuaqinggan Shisanwei Wan | Clearing liver heat and detoxifying | It is used for liver function decline, poisoning, "Yama" disease, lumbar and renal injury, frequent urination and blood urination. It is especially effective for eye diseases caused by blood fever |
| HuangBaiBaWei Pill | Clearing heat, cooling blood and consolidating essence | It is used for kidney fever, urinary tract infection, blood in urine and excessive menstruation |
| Jixiang Ankun Wan | Regulating menstruation and activating blood circulation, replenishing qi and calming nerves | For irregular menstruation, postpartum fever, anxiety of mind, dizziness and headache, weakness of waist and knee, edema of limbs and breast swelling |
| Naru Sanwei Wan | Eliminating "Stickiness", dehumidify, dispelling wind, relieving pain and dispersing cold | For rheumatism, joint pain, cold pain in waist and legs, toothache, diphtheria and other diseases |
| Nuangong Qiwei Wan（Powder） | Regulating menstruation and nourishing blood, warming uterus and stoping leucorrhea | For heart and kidney "Heyi" disease, qi stagnation, low back pain, cold pain in the lower abdomen, irregular menstruation and excessive leucorrhea |
| Qinggan Jiuwei Wan | Eliminating "Stickiness", relieving fever and cough | For plague fever, influenza, cough, sore throat. |
| Qingre Bawei Wan（Powder、Capsule） | Clearing away heat and toxic material | It is used for blazing, blood heat, visceral heat, lung heat, cough, blood in sputum, liver fire and rib pain |
| Roukou Wuwei Wan | Dispelling heart "Heyi" disease | For irritability, insomnia and uneasiness; It is especially effective for heart "Heyi" disease |
| Surilao Qingfei Zhike Capsule | Clearing lung heat, relieving cough and eliminating phlegm | It is used for bloody pulmonary fever, acute tracheobronchial bronchitis and acute attack of chronic bronchitis |
| Diaoyuandabu Ershiwuwei Tangsan | Convergence, detoxification, regulating cold and heat | It is used for long-term illness, fatigue, dry mouth, loss of appetite and epigastric pain |
| Wulan Shisanwei Tangsan | Removing heat from blood | It is used for excessive blood heat, headache, red eyes and hypertension |
| Xiaoji Jiebai Wan | Dissipating cold in the warm, eliminating food stagnation and relieving pain | It is used for internal stagnation of the food, fullness, swelling,pain,and indigestion due to deficiency and cold in the middle Jiao |
| Yishen Shiqiwei Wan | Clearing kidney heat, eliminating "Stickiness" and consolidating essence | It is used for kidney cold and kidney heat, waist and knee pain, nocturnal ejaculation, testicular swelling |
| Xiaoer Shikou Powder | Quelling the “Badagan Xieri” and eliminating stickiness and poison | It is used for increasing stool frequency with yellow green water, undigested food and milk, and some foam or mucus, abdominal pain, abdominal distention, nausea, vomiting, thirst, white or yellow fur, white or yellow urine, less volume, slow, deep or thin pulse due to light and medium-sized children with rotavirus enteritis |
| Zhachong ShisanweiWan | Dispeling wind, dredging orifices, relaxing tendons and activating blood circulation, calm mind and nerves, eliminating "Xieri Wuzhai" | It is used for hemiplegia, left paralysis, right paralysis, crooked mouth and eyes, numbness of limbs, unfavorable waist and legs, unclear speech, muscle and bone pain, nerve paralysis, rheumatism and joint pain |
| Zhenbao Wan | Clearing heat, calming nerves, relaxing muscles and activating collaterals, eliminating "xieriwusu" | For nervous system diseases, hemiplegia, rheumatism, rheumatoid, muscle atrophy, nerve paralysis, kidney damage, pulse injury, plague fever, long-term cure and other diseases |
| Zhenzhu tongluo Wan | Cleaning the heat,opening the oifice and drying the yellow water | It is used for rheumatoid, nephropathy, pulse disease, hemiplegia and hemiplegia |
| Liangxue Shiwei Powder（Pill） | Cooling blood and brighting eyes | For liver fire, lung heat, headache and red eyes |
| Aiweixin oral solution | Dredging collaterals and blood, strengthening the heart and brain, and the dominant organs | For chest pain, palpitation, insomnia and forgetfulness. Arrhythmia, neurasthenia |
| Baixuanxiatare Pill（Capsule） | Removing abnormal mucus and bile fluid, reducing swelling and relieving itching | It is used to treat tinea manus, tinea pedis, tinea versicolor, psoriasis, allergic dermatitis and acne |
| Compound gaoziban pills | Strengthening the heart and brain, calming the mind and dredging the pulse | For palpitation, insomnia, dizziness, headache, neurasthenia, hypertension, etc |
| Compound Muniziqi Granule | Regulating body fluid | They are four kinds of abnormal humoral maturation agents |
| Hanchuan Zupa Granule | Preventing cough, resolving phlegm, warming lung and relieving asthma | For acute cold, cough caused by cold evil and abnormal mucinous asthma |
| Zukamu Granule | Regulating abnormal body fluid, clearing away heat,inducing perspiration and dredging orifices | For cold, cough, fever, no sweat, sore throat, stuffy nose and runny nose |
| Hugan Buzure Granule | Tonifying liver and stomach, dispersing Qi and relieving pain, benefiting gallbladder and water | It is used for liver cold, stomach pain, spleen hypochondriac pain, joint bone pain, rheumatism and urinary system diseases |
| Jianxin hemilgaozi ban'an pirated tablets | Tonifying the control organs | It is ued for palpitation, insomnia and dreaminess |
| Luobufukebiri Wan | Warming brain and kidney, benefiting heart and filling essence | For impotence, depression, spermatorrhea, premature ejaculation, body deficiency, weight loss, neurasthenia |
| Mamulan antidiarrheal capsule | Clearing septicemia, degrading abnormal bile fluid, and stoping diarrhea | It is used for abdominal pain, diarrhea, nausea and indigestion |
| Meiguihua oral solution | Tonifying the control organs（heart、brain、liver） | For palpitation, shortness of breath, stomach pain, vomiting, limb paralysis pain, mental fatigue |
| Niaotongkakenaiqi Pill | Relieving the pain ,diuresis | For pain in urination, endless urination, blood in urination, urethral pus, etc |
| Qingre Carson Granule | Clearing liver heat, dredging gallbladder,strengthening stomach,helping digestion, promoting diuresis and eliminating edema | It is used for damp heat of bottom pour, yellow bile, stomach pain, less food, less edema and urine. |
| Pomegranate blood tonifying syrup | Invigorating blood and brain | It is used for anemia, palpitation, shortness of breath, anxiety and dizziness caused by excessive abnormal biliary fluid |
| Tongzhisurunjiang Capsule（Pill） | Opening block, eliminating edema and relieving pain | For arthralgia, rheumatism, rheumatoid arthritis, sciatica |
| Yanxiao Dinar syrup | Promoting diuresis, eliminating edema, reducing fever and relieving pain | For all kinds of hepatitis, cholecystitis, urinary tract infection, etc |
| YangXinDaWaYiMiXiKeMiGao | Invigorating the stomach and refreshing the mind | For chest pain, palpitation, stomach deficiency, asthenia and neurasthenia |
| Yixin badiranjibuya Granule | Tonifying the heart and brain, helping diuresis and stoping asthma | It is used for mental fatigue, insomnia, upset, asthma and neurasthenia |
| YajiaoHadun San | Clearing heat and detoxification, relieving pain and hemostasis | For cold, fever, laryngitis, chest and abdominal pain, fatigue, palpitation, irregular menstruation, postpartum bleeding |
| HufengJiu | Eliminating Wind and removing Dampness | Arthralgia caused by rheumatic obstruction, with joint pain and heavy limbs; Acute rheumatism and rheumatoid arthritis see the above symptoms |
| Duyiwei Capsule(pill) | Promoting blood circulation, relieving pain, removing blood stasis and stoping bleeding | It is used for knife edge pain, bleeding, trauma, fracture, muscle and bone sprain, rheumatism and arthralgia, metrorrhagia, dysmenorrhea, gum swelling and bleeding after a variety of surgical operations |
| Anerning Granule | Clearing heat and dispelling wind, transforming diseases and relieving cough | It is used for children with wind heat, cold, cough, phlegm, fever, sore throat, upper respiratory tract infection, etc |
| Honghua Ruyi Wan | Dispelling wind, relieving pain, regulating menstruation, removing spots | It is used for women's anemia, rheumatism, vaginitis, cervical erosion, upset and blood deficiency, irregular menstruation, dysmenorrhea, lower limb joint pain, muscle and bone swelling, morning stiffness, numbness, cold pain in lower abdomen and cold dampness arthralgia |
| Dengzhan Shengmai Capsule | Supplementing qi and nourishing Yin, promoting blood circulation and brain | Chest arthralgia and heartache caused by deficiency of Qi and Yin, blood stasis blocking brain collaterals;Sequelae of stroke,;Dementia,;Amnesia and numbness of hands and feet; Coronary heart disease, Angina pectoris, Ischemic cardiovascular and cerebrovascular disease and hyperlipidemia |
| Yindan Xinnaotong Capsule | Promoting blood circulation and removing blood stasis, promoting qi and relieving pain, and eliminating food stagnation | Chest arthralgia caused by qi stagnation and blood stasis, including chest pain, chest tightness, shortness of breath, palpitation, etc; Coronary heart disease, angina pectoris, hyperlipidemia, cerebral arteriosclerosis, stroke and stroke sequelae |
| Lingdancao Granule | Clearing heat and expelling wind, detoxifying and benefiting pharynx, relieving cough and removing phlegm | Wind heat toxin, sore throat and lung heat cough; Acute pharyngitis, tonsillitis and upper respiratory tract infection with the above symptoms |

**Table S2**

| Proposed search strategy to search MEDLINE (PubMed) | | |
| --- | --- | --- |
|  | Search terms | |
| #1 | ((((((((((((((((((((((((((((((((((((((((((((((Ershiwuwei Songshi[Title/Abstract]) OR (Ershiwuwei Shanhu[Title/Abstract])) OR (Shiyiwei Nengxiao[Title/Abstract])) OR (Shi’erwei Yishou[Title/Abstract])) OR (Shisanwei bangga[Title/Abstract])) OR (ShiWuWeiChenXiang[Title/Abstract])) OR (Seventy-flavor Zhenzhu[Title/Abstract])) OR (QiWeiTieXie[Title/Abstract])) OR (Bawei Chenxiang[Title/Abstract])) OR (Jiuwei Shihuihua[Title/Abstract])) OR (WuWeiSheXiang[Title/Abstract])) OR (Renqing Mangjue[Title/Abstract])) OR (Renqing Changjue[Title/Abstract])) OR (LiuWeiAnXiao[Title/Abstract])) OR (JieBaiWan[Title/Abstract])) OR (Cuitang[Title/Abstract])) OR (Changsong Bawei Chenxiang[Title/Abstract])) OR (DaYueJing[Title/Abstract])) OR (ErShiWeiChenXiang[Title/Abstract])) OR (ErShiWeiRouDouKou[Title/Abstract])) OR (ErShiWuWeiDaTang[Title/Abstract])) OR (ErshiwuweiErCha[Title/Abstract])) OR (ErShiWuWeiLvXue[Title/Abstract])) OR (Ershiwuwei zhenzhu[Title/Abstract])) OR (Jiuwei Niuhuang[Title/Abstract])) OR (Lishukang[Title/Abstract])) OR (LiuGanWan[Title/Abstract])) OR (LiuWeiNengXiao[Title/Abstract])) OR (NuoDiKang[Title/Abstract])) OR (Pazhu[Title/Abstract])) OR (QiWeiHongHuaShuSheng[Title/Abstract])) OR (Ruyi Zhenbao[Title/Abstract])) OR (Thirty five flavor Chenxiang[Title/Abstract])) OR (ShanHuQiShiWei[Title/Abstract])) OR (ShiWeiDiDa[Title/Abstract])) OR (ShiWeiHeiBingPian[Title/Abstract])) OR (Shiwei Longdanhua[Title/Abstract])) OR (Shiwuwei Heiyao[Title/Abstract])) OR (ShiWuWeiLongDanHua[Title/Abstract])) OR (Pomegranate Jianwei[Title/Abstract])) OR (ZhituoJiebai[Title/Abstract])) OR (Zuozhu Daxi[Title/Abstract])) OR (AnShenWan[Title/Abstract])) OR (Liuwei Mingmu[Title/Abstract])) OR (Duyiwei[Title/Abstract])) OR (An'erning[Title/Abstract])) OR (Honghua Ruyi[Title/Abstract]) | Tibetan medicine  (藏药) |
| #2 | (((((((((((((((((((((((((((((((((((((ShiLiuWeiDongQing[Title/Abstract]) OR (Qiwei Guangzao[Title/Abstract])) OR (QiWeiPuTao[Title/Abstract])) OR (QingXinChenXiangBaWei[Title/Abstract])) ) OR (BaWeiQingXinChenXiang[Title/Abstract])) OR (Bawei Tanxiang[Title/Abstract])) OR (SanZiSan[Title/Abstract])) OR (Sanwei tribulus[Title/Abstract])) OR (WuWeiShaJi[Title/Abstract])) OR (WuweiQingzhuo[Title/Abstract])) OR (Liuwei Muxiang[Title/Abstract])) OR (Siweitumuxiang[Title/Abstract])) OR (Alatan Wuwei[Title/Abstract])) OR (Anshen Buxin Liuwei[Title/Abstract])) OR (BaTeRiQiWei[Title/Abstract])) OR (DaHuangSanWei[Title/Abstract])) OR (FengShiErShiWuWei[Title/Abstract])) OR (Hanshuishi Ershiyiwei[Title/Abstract])) OR (HongHuaQingGanShiSanWei[Title/Abstract])) OR (HuangBaiBaWei[Title/Abstract])) OR (Jixiang Ankun[Title/Abstract])) OR (NaRuSanWei[Title/Abstract])) OR (NuanGongQiWei[Title/Abstract])) OR (Qinggan Jiuwei[Title/Abstract])) OR (Qingre Bawei[Title/Abstract])) OR (Roukou Wuwei[Title/Abstract])) OR (Surilao Qingfei Zhike[Title/Abstract])) OR (DiaoYuanDaBuErShiWuWei[Title/Abstract])) OR (WuLanShiSanWei[Title/Abstract])) OR (Xiaoji Jiebai[Title/Abstract])) OR (Xiao Er Shi Kou[Title/Abstract])) OR (YiShenShiQiWei[Title/Abstract])) OR (ZhaChongShiSanWei[Title/Abstract])) OR (ZhenBaoWan[Title/Abstract])) OR (ZhenZhuTongLuo[Title/Abstract])) OR (Liangxue Shiwei[Title/Abstract])) OR (LiuWeiAnXiao[Title/Abstract]) | Mongolian medicine  (蒙药) |
| #3 | ((((((((((((((((((((Aiweixin[Title/Abstract]) OR (baixuanxiatare[Title/Abstract])) OR (FuFangGaoZiBan[Title/Abstract])) OR (compound gaozaban[Title/Abstract])) OR (Compound muniziqi[Title/Abstract])) OR (hanchuan zupa[Title/Abstract])) OR (Hugan buzure[Title/Abstract])) OR (Jianxin hemilgaozi ban'an pirated[Title/Abstract])) OR (LuoBuFuKeBiRi[Title/Abstract])) OR (mamula antidiarrheal[Title/Abstract])) OR (Meiguihua oral solution[Title/Abstract])) OR (NiaoTongKaKeNaiQi[Title/Abstract])) OR (qingke carson[Title/Abstract])) OR (pomegranate bloodtonifying syrup[Title/Abstract])) OR (Tongzhisurunjiang[Title/Abstract])) OR (XiPaYiGuZuoYe[Title/Abstract])) OR (yan xiao dinar syrup[Title/Abstract])) OR (YangXinDaWaYiMiXiKeMiGao[Title/Abstract])) OR (Yixin badiranjibuya[Title/Abstract])) OR (Zukamu[Title/Abstract]) | Uighur medicine  (维药) |
| #4 | [(YajiaoHadun[Title/Abstract]) OR (Dengzhan shengmai[Title/Abstract])](https://pubmed.ncbi.nlm.nih.gov/?term=(YajiaoHadun%5bTitle/Abstract%5d)+OR+(dengzhanshengmai%5bTitle/Abstract%5d)&page=1) | Dai medicine  (傣族药) |
| #5 | (HufengJiu[Title/Abstract]) | Jingpo medicine  (景颇族药) |
| #6 | (Yindan Xinnaotong[Title/Abstract]) | Miao medicine  (苗药) |
| #7 | (Lingdancao[Title/Abstract]) | Yi medicine  (彝药) |
| #8 | #1 OR #2 OR #3 OR #4 OR #5 OR #6 OR #7 | |

**Table S3**

Market prices of Chinese traditional ethnic patent medicine

| Drug Name | Oral Single dose/ Time | Frequency | Market unit price (Yuan) / g (ml) | Market price (Yuan) / day |
| --- | --- | --- | --- | --- |
| Ershiwuwei Songshi Wan | 1g | qd | 3.5-18 | 3.5-18 |
| Ershiwuwei Shanhu Wan | 1g | qd | 3.2-35.8 | 3.2-35.8 |
| Shiyiwei Nengxiao Wan（Capsule） | 0.6-0.9g | bid | 3.1-18.9 | 3.7-34 |
| Shi’erwei Yishou Powder | 1g | bid | 4.5-9.5 | 9-19 |
| Shisanwei Bangga Powder | 1-1.5g | bid | - | - |
| Shiwuwei Chenxiang Wan | 3-4g | bid | 1.1-6.4 | 6.6-51.2 |
| Seventy-flavor Zhenzhu Wan | 1g | Serious patients take 1g a day, and moderate patients take 1g for 3-7 days | 47.5-93.3 | 47.5-93.3（Severe）  6.8-31.1（Moderate） |
| Qiwei Tiexie Wan | 1g | bid | 1-3.4 | 2-6.8 |
| Bawei Chenxiang Powder(Pill、Capsule、Wan） | 1.8-3g(Pill）  1.2g(Capsule）  1-1.5g（Wan） | bid-tid | 4.7-8.8（Wan） | - |
| Jiuwei Shihuihua Powder | 0.6-0.9g | bid | - | - |
| Wuwei Shexiang Wan | 0.06-0.09g | qd | 13.6-125.9 | 0.8-11.3 |
| Renqing Mangjue | 1-1.5g | qd | 17.8-66.7 | 17.8-100.1 |
| Renqing Changjue | 1g | Serious patients take 1g every three, seven or ten days | 44.2-499.5 | 44.2-499.5(Severe）  6.3-166.5（Moderate） |
| Liuwei Anxiao Powder（Tibetan/Mongolian medicine） | 1.5-3g | bid-tid | 0.5-2.2(Wan）  0.4-2.8（Capsule）  0.6-3.2（Powder） | 1.5-19.8（Wan）  1.2-25.2（Capsule）  1.8-28.8（Powder） |
| Jiebai Wan | 0.8g | bid-tid | 1-5.2 | 1.6-12.5 |
| Cuitang Wan | 4-8g | tid | - | - |
| Changsong Bawei Chenxiang Powder | 1.3-2g | bid-tid | 2.5-5.3 | 6.5-31.8 |
| Dayuejing Wan | 1.8-3g | tid | 2.9-6.2 | 15.7-55.8 |
| Ershiwei Chenxiang Wan | 3-4g | bid | 3.0-5.9 | 18-47.2 |
| Ershiwei Roudoukou Wan | 2.25-3g | bid | 1-3.7 | 4.5-22.2 |
| Ershiwuwei Datang Wan | 1-1.5g | tid | 1.5-2.8 | 4.5-12.6 |
| Ershiwuwei ErCha Wan | 1.2-1.5 | bid-tid | 3.3-7.3 | 8-32.9 |
| Ershiwuwei Lvxue Wan | 0.75g | bid-tid | 1.7-33.8 | 2.6-76.1 |
| Ershiwuwei zhenzhu Wan | 1g | qd-bid | 2.3-18.3 | 2.3-36.6 |
| Jiuwei Niuhuang Wan | 2-2.5g | tid | 1.6-4.8 | 9.6-36 |
| Lishukang Capsule | 1g | tid | 2.4-5.5 | 7.2-16.5 |
| LiuGanWan | 1-2g | bid-tid | 0.3-4.3 | 0.6-25.8 |
| LiuWei Nengxiao Wan（Capsule） | 1.5-2g；0.45-0.9g | bid;tid | 0.5-2.5(Wan）  2.1-4.8（Capsule） | 1.5-10(Wan）  2.8-13（Capsule） |
| Nuodikang Pill（Capsule、Granule、Oral solution） | 0.28-0.56g(Capsule）  5g（Granule）  10ml（oral solution） | tid | 2.9-5.5（Capsule）  2.1-4.4（Granule）  0.4（oral solution） | 2.4-9.2(Capsule）  31.5-66（Granule）  12（oral solution） |
| Pazhu Wan(Capsule） | 0.9g | qd | 4.25-12.6 | 3.8-14 |
| Qiwei Honghuashusheng Powder（Pill） | 1.2-1.8g | bid | 2.7-4.9（Wan） | 6.5-17.64 |
| Ruyi Zhenbao Wan | 2-2.5g | bid | 2.7-7 | 10.8-35 |
| Thirty five flavor Chenxiang Wan | 3-4g | bid | 5.3-6.4 | 31.8-51.2 |
| ShanHuQiShiWei Wan | 每time1g | - | 23.8-49.7 | 23.8-49.7 |
| Shiwei Dida Capsule | 0.9g | tid | 4.4-6 | 11.8-16.2 |
| Shiweiheibingpian Wan | 2-3g | bid | 3.4-7.0 | 13.6-42 |
| Shiwei Longdanhua Granule（Capsule） | 1.35g；3g | tid | 0.83-1.7(Granule）  1.8-4.3（Capsule） | 3.4-15.3(Granule）  7.3-38.7（Capsule） |
| Shiwuwei Heiyao Wan | 1.6-2.4g | bid | 1.25-3.1 | 4-14.9 |
| ShiWuWeiLongDanHua Wan | 1.8-2.4g | tid | 1-6.6 | 5.4-47.5 |
| Pomegranate Jianwei Wan(Pill、Capsule、Powder） | 1.2-1.8g（Wan）  1.2g（Pill）  0.9g（Capsule）  1.2g（Powder） | bid-tid（Wan、Pill、Capsule）  qd-tid(Powder） | 2.6-5.2（Wan）  1.7-4.0（Pill）  2.2-7.2（Capsule）  2.8-5.5（Powder） | 6.2-28.1(Wan）  4.1-21.6（Pill）  5.3-38.9（Capsule）  3.4-29.7（Powder） |
| ZhituoJiebai Wan | 2.8g-4.2g | tid | 1.2-1.5 | 10.1-18.9 |
| Zuozhu Daxi | 1g | 2-3 day/1g | 4.8-17.8 | 1.6-8.9 |
| Anshen Wan | 0.6-0.9g | bid | 3.9-10.1 | 4.7-18.2 |
| Liuwei Mingmu Wan | 1.5g | bid | 6.2-8.2 | 18.6-24.6 |
| Shiliuwei Dongqing Wan | 6g | qd-bid | - | - |
| Qiwei Guangzao Wan | 7g | qd-bid | - | - |
| Qiweiputao Powder | 3g | qd-bid | - | - |
| Qingxinchenxiang Bawei Powder/Bawei Qingxinchenxiang Powder | 3g | qd-bid | 0.9-3.2(Powder）  0.1-3.3（Wan） | 2.7-19.2(Powder）  0.3-19.8（Wan） |
| Bawei Tanxiang Powder | 2-3g | qd-bid | - | - |
| Sanzisan | 3-4.5g | bid-tid | 2.4（Powder）  0.8-1.4（Granule） | 14.4-32.4（Powder）  4.8-18.9（Granule） |
| Sanwei tribulus Powder | 3-4.6g | bid-tid | - | - |
| Wuwei Shaji Powder | 3g | qd-bid | 0.4-0.7(Granule） | 1.2-4.2(Granule） |
| WuweiQingzhuo Powder | 2-3g | qd-bid | 1.7-2.8(Wan）  0.8-8（Powder） | 3.4-16.8(Wan）  1.6-48（Powder） |
| Liuwei Muxiang Powder | 2-4g | qd-bid | 0.9-2.1（Capsule） | 1.8-16.8(Capsule） |
| Siwei Tumuxiang Powder | 2.5-3.6g | bid-tid | - | - |
| Alatan Wuwei Wan | 2.2-3g | qd-bid | 0.8-3.1 | 1.8-18.6 |
| Anshen Buxin Liuwei Wan | 2.2-4g | qd-bid | 11.7-21.3 | 25.7-170.4 |
| Bateri Qiwei Wan | 1.8-2.6g | qd-bid | 0.6-3.2 | 1.08-16.6 |
| Dhuang Sanwei Pill | 0.3-0.9g | bid-tid | 7.6-24.4 | 4.6-65.9 |
| FengShi Ershiwuwei Wan | 2.2-3g | qd-bid | 6.3-41.2 | 13.9-247.2 |
| Hanshuishi Ershiyiwei Powder | 1.5-3g | qd-bid | 3.8-4.6 | 5.7-27.6 |
| Honghuaqinggan Shisanwei Wan | 2.5-3g | qd-bid | 0.75-5.3 | 1.9-31.8 |
| HuangBaiBaWei Pill | 1.5-3g | bid-tid | 1.8-5.1 | 5.4-45.9 |
| Jixiang Ankun Wan | 2.2-3g | qd-bid | 1.2-8 | 2.6-48 |
| Naru Sanwei Wan | 0.6-1g | qd | 1.25-13.8 | 0.75-13.8 |
| Nuangong Qiwei Wan（Powder） | 2.2-3g | qd-bid | 0.7-2.7（Wan）  1.5-3.3（Powder） | 1.5-16.2 |
| Qinggan Jiuwei Wan | 1.8-2.6g | qd | 0.41-4 | 0.7-10.4 |
| Qingre Bawei Wan（Powder、Capsule） | 1.8-3g | qd-bid | 4.25-4.5（Wan）  1.8-2.9（Powder）  7.6-13.2（Capsule） | 7.7-27(Wan）  3.2-17.4（Powder）  13.7-79.2（Capsule） |
| Roukou Wuwei Wan | 1.8-3g | qd-tid | 0.8-2.1 | 1.44-18.9 |
| Surilao Qingfei Zhike Capsule | 1.2g | tid | 0.1-4.1 | 0.36-14.8 |
| Diaoyuandabu Ershiwuwei Tangsan | 1.5-3g | qd-tid | 2.7-3.3 | 4.1-29.7 |
| Wulan Shisanwei Tangsan | 3-5g | qd-tid | 3.2 | 9.6-48 |
| Xiaoji Jiebai Wan | 1.8-9g | qd-bid | 0.43-3 | 0.8-54 |
| Yishen Shiqiwei Wan | 1-2.2g | qd | 1.5-12 | 1.5-26.4 |
| Xiaoer Shikou Powder | 1-2g | bid/tid | - | - |
| Zhachong ShisanweiWan | 1-2g | qd | 3.5-17.5 | 3.5-35 |
| Zhenbao Wan | 2.6-3g | qd-bid | 1.3-6.7 | 3.4-40.2 |
| Zhenzhu tongluo Wan | 1.8-2.6g | qd-bid | 3.8-10 | 6.8-52 |
| Liangxue Shiwei Powder（Pill） | 1.5-3g | qd-bid | - | - |
| Aiweixin oral solution | 10ml | bid | 0.8-1.5 | 16-30 |
| Baixuanxiatare Pill（Capsule） | 1.2g-2g | tid | 1.1-4.7(Pill）  4.4-8.4（Capsule） | 4.0-28.2（Pill）  15.8-50.4（Capsule） |
| Compound gaoziban pills | 4-6Pill | bid | 1.5/Pill | 12-18 |
| Compound Muniziqi Granule | 6g | tid | 0.4-1.1 | 7.2-19.8 |
| Hanchuan Zupa Granule | 6g | bid | 0.2-1.1 | 2.4-13.2 |
| Zukamu Granule | 12g | tid | 0.1-0.8 | 3.6-28.8 |
| Hugan Buzure Granule | 6g | tid | 0.7-1.4 | 12.6-15.2 |
| Jianxin hemilgaozi ban'an pirated tablets | 1g | bid | - | - |
| Luobufukebiri Wan | 3-4.5g | bid | 5.5-5.8 | 33-52.2 |
| Mamulan antidiarrheal capsule | 0.9g | tid | 4.9-10.8 | 13.2-29.2 |
| Meiguihua oral solution | 10ml | tid | 1.84/ml | 55.2 |
| Niaotongkakenaiqi Pill | 1.5-2.5g | bid | 3.8-5.2 | 11.4-26 |
| Qingre Carson Granule | 6g | tid | 0.6-1.3 | 10.8-23.4 |
| Pomegranate blood tonifying syrup | 20-30ml | tid | 0.2-0.7 | 12-63 |
| Tongzhisurunjiang Capsule（Pill） | 1.25-1.75g | bid | 2.1-11.5(Pill）  1.3-7.9（Capsule） | 5.3-40.3（Pill）  3.3-27.7（Capsule） |
| Yanxiao Dinar syrup | 30ml | tid | 0.3-0.49/ml | 27-44.1 |
| YangXinDaWaYiMiXiKeMiGao | 3g | bid | 3.1-7.1 | 18.6-42.6 |
| Yixin badiranjibuya Granule | 3g | tid | 0.1-1.4 | 0.9-12.6 |
| YajiaoHadun San | 3-9g | tid | - | - |
| HufengJiu | 15-20ml | bid | 0.2-0.3 | 6-12 |
| Duyiwei Capsule(pill) | 0.3/0.5g | tid | 0.5-2.4（Pill）  0.7-3.9（Capsule）  0.4-7.7（Wan）  0.4-1.4（Granule） | 0.45-3.6（Pill）  0.63-5.9（Capsule）  0.36-11.6（Wan）  0.36-2.1（Granule） |
| Anerning Granule | 1.5-6g | tid | 1.3-1.8 | 5.9-32.4 |
| Honghua Ruyi Wan | 1.2g | bid | 1.5-3.7 | 3.6-8.9 |
| Dengzhan Shengmai Capsule | 0.36g | tid | 7.4-14.8 | 8.0-16.0 |
| Yindan Xinnaotong Capsule | 0.8-1.6g | tid | 1.6-2.6 | 3.8-12.5 |
| Lingdancao Granule | 3-6g | 3-4time | 0.8-7.4 | 7.2-177.6 |

**Table S4**

The composition of 105 Traditional Chinese ethnic patent medicines

| Traditional Chinese ethnic patent medicines | Drug composition |
| --- | --- |
| Ershiwuwei Songshi Wan | Turquoise、pearl、Coralline、Cinnabar、Terminalia chebula、Iron powder、emblic leafflower fruit、trogopterus dung cream、Sandalwood、lignum dalbergiae odoriferae、elecampaneAristolochia debilis、Adhatoda vasica Nees、Calculus bovis、广elecampane、herba meconopsis、Aconitum napellus、Myristica fragrans Houtt、Clove、Saxifraga stolonifera、Terminalia chebula、Tabasheer、Stigma Croci、kapok、musk、 travertine |
| Ershiwuwei Shanhu Wan | Coralline、pearl、Lapis lazuli、Mother of pearl、Terminalia chebula、elecampane、Carthamus tinctorius、Clove、agilawood、Cinnabar、keel、calamine、yellow croaker ear-stone、magnetite、red claystone、Sesamum indicum、gourd、Tatarian aster、Herba Swertiae Mussotll、Tibetan calamus、bangna、Flos Pyrethri Tatsienensis、 liquorice、Stigma Croci、Calculus Bovis Artifactus |
| Shiyiwei Nengxiao Wan（Capsule） | Tibentan elecampane、Lobular lotus、Dried ginger、Seabuckthorn cream、Terminalia chebula（denucleate）、Snake meat、Rhubarb、Fanghai、North Hanshuishi (Calcined)、sal ammoniac、Alkali flowers |
| Shi’erwei Yishou Powder | hooker winghead root 、bangga、erect hypecoum herb 、Tibetan crazyweed 、Aconitum balfourii Stapf leaf、 travertine、Calculus bovis、musk、benzoin、trogopterus dung cream、Sandalwood、Carthamus tinctorius |
| Shisanwei Bangga Powder | bangga、Semen Herpetospermi 、Gentiana siphonantha、Herba Swertiae Mussotll、Herba corydalis impatiens、Ixeris chinensis nakai、Herba Lagotis 、cortex berberidis、erect hypecoum herb 、Chrysosplenium nudicaule Bge、Calculus bovis、Carthamus tinctorius、conessi |
| Shiwuwei Chenxiang Wan | agilawood、elecampane、Sandalwood、Sandalwood、Carthamus tinctorius、Myristica fragrans Houtt、Mountain horseradish、Raspberries stem（Remove the skin and heart）、Polygonum aubertii Henry （Remove the skin）、Arum sagittifolium 、 travertine、 fructus choerospondiatis 、Terminalia chebula（denucleate）、erminalia chebula（denucleate）、emblic leafflower fruit |
| Seventy-flavor Zhenzhu Wan | pearl、Sandalwood、lignum dalbergiae odoriferae、 liquorice、Tabasheer、Stigma Croci、Calculus bovis in vitro、Calculus Bovis Artifactus、Coralline、carnelian、Nine eyes stone、zuotai etc. |
| Qiwei Tiexie Wan | Iron powder（processed by Terminalia chebula）、 gypsum rubrum （processed by milk）、elecampane、elecampane、Herba Dracocephali Tangutici 、Carthamus tinctorius、trogopterus dung cream |
| Bawei Chenxiang Powder(Pill、Capsule、Wan） | agilawood、Myristica fragrans Houtt、fructus choerospondiatis 、 travertine、libanotus、elecampane、Terminalia chebula、kapok |
| Jiuwei Shihuihua Powder | travertine、Calculus bovis、bangga、Mountain horseradish、Herba Lagotis 、Carthamus tinctorius、Herba Rhodiolae、 liquorice(remove the peel)、Sandalwood |
| Wuwei Shexiang Wan | musk、Terminalia chebula（denucleate）、Aconitum balfourii Stapf 、elecampane、Tibetan calamus |
| Renqing Mangjue | lignum dalbergiae odoriferae、agilawood、Terminalia chebula(denucleate)、Tabasheer、Stigma Croci、Sandalwood、Calculus bovis in vitro、Calculus Bovis Artifactus、bear gall、amber、Turquoise、Zuotai etc. |
| Renqing Changjue | 由pearl、Cinnabar、Sandalwood、lignum dalbergiae odoriferae、agilawood、Terminalia chebula、Calculus bovis、musk、Stigma Croci etc. |
| Liuwei Anxiao Powder（Tibetan/Mongolian medicine） | Tibetan elecampane、Rhubarb、resurrection lily rhizome、North Hanshuishi (Calcined)、Terminalia chebula、Alkali flowers |
| Jiebai Wan | Terminalia chebula，gypsum rubrum，hooker winghead root ，trogopterus dung cream，elecampane，seed of pomegranate，fructus chaenomelis lagenariae，agilawood，Clove， travertine，Carthamus tinctorius，Myristica fragrans Houtt，Alpinia katsumadai，Semen tsaoko |
| Cuitang Wan | Tibetan Elecampane cream、Tibetan elecampane、Raspberries stem（Remove the peel and heart）、Wide tendon vine (Remove the peel)、Dried ginger、Terminalia chebula、emblic leafflower fruit、Terminalia chebula(denucleate）、younghusband jerusalemsage root |
| Changsong Bawei Chenxiang Powder | agilawood、fructus choerospondiatis 、Sandalwood、lignum dalbergiae odoriferae、Myristica fragrans Houtt、Tabasheer、Carthamus tinctorius、The seed radish |
| Dayuejing Wan | gypsum rubrum 、Tabasheer、Carthamus tinctorius、Myristica fragrans Houtt、Amomum tsao-ko 、cardamun、Clove、Terminalia chebula、emblic leafflower fruit、Sandalwood、lignum dalbergiae odoriferae、elecampane、long pepper、seed of pomegranate 、conessi 、Semen Herpetospermi 、nuces vomicae、Tibetan elecampane、benzoin、Slag breaking cream、iron powder 、bangga、Herba Swertiae Mussotll、Lagotis glauca 、Herba corydalis impatiens、muscicolous woodbetony root 、 Herba Dracocephali Tangutici 、herba meconopsis、Rhubarb、dandelion、calamine、Ouqu、Bear bile powder、Calculus bovis、Calculus Bovis Artifactus |
| Ershiwei Chenxiang Wan | agilawood、Clove、fructus chaenomelis lagenariae、Myristica fragrans Houtt、Carthamus tinctorius、fructus choerospondiatis 、Tibetan elecampane、 travertine、cornu cervi、libanotus、Mother of pearl、elecampane、nuces vomicae、Terminalia chebula、Lagotis glauca 、kapok、emblic leafflower fruit、lignum dalbergiae odoriferae、Rabbit heart、Artificial Calculus bovis |
| Ershiwei Roudoukou Wan | Nutmeg, Parmesan, aloes, Travertine, Jujube, safflower, Fennel, clove, garlic (charcoal), cardamom, asafula, grass fruit, terminalia chebula, Frankincense, terminalia chebula, catechu tea, polygamous fruit, Ricartto, sandalwood,calculus bovis factitius |
| Ershiwuwei Datang Wan | Safflower、Terminalia chebula、Terminalia terminalia、Emblic leafflower fruit (nuclear)、Tibet inula root、elecampane、Herpetospermum caudigerum Wall、faeces ochotonae erythrotis、Pomegranate seeds、cardamom、 Fructus Chaenomelis、Pig blood powder、Dracocephalum tanguticum Maxim、Rhizoma drynariae、Coriander、Swertia bimaculata、Lagotis integra、Gentiana siphonantha、Bangga、erect hypecoum、Tatarian aster、Herba gentianae urnulae、Herba gentianae urnulae、Ramulus myricariae、Herba corydalis impatiens |
| Ershiwuwei ErCha Wan | catechu、Terminalia chebula、Terminalia terminalia、Emblic leafflower fruit 、Tiben pleurospermum uralense、rhizoma polygonati、Asparagus cochinchinensis、Mirabilis japonicum、Tribulus teristus、Frankincense、Cassia seeds、Ambrette、Tinospora cordifolia、Piper longum、Iron powder、faeces ochotonae erythrotis、Iron hammer、Artificial musk、Tibetan calamus、elecampane、Buffalo horn、Mother of pearl、Gansu Oxytropis、Flat thorn rose、Gentiana siphonantha |
| Ershiwuwei Lvxue Wan | Donkey blood、Rhamnella gilgitica Mansf. et Molch、dalbergia wood、sandalwood、Terminalia terminalia、Terminalia chebula、travertine、Emblic leafflower fruit 、Myristica fragrans Houtt、Cloves、Amomum 、cardamom、Cassia seeds、Frankincense、kapok、Ambrette、Herba pterocephali、Gentian、Rosette herb、Herba corydalis impatiens、Tinospora cordifolia、cortex fraxini、Artificial musk、Safflower、calculus bovis factitius |
| Ershiwuwei zhenzhu Wan | pearl、Mother of pearl、Myristica fragrans Houtt、travertine、Safflower、Amomum 、Cloves、dalbergia wood、cardamom、Terminalia chebula、sandalwood、Emblic leafflower fruit 、agilawood、cinnamon、Terminalia terminalia、crab、elecampane、Fructus Malvae Vertillatae、Piper longum、Strawberry seedlings、Vermiculite Schist seu、Buffalo horn、Vermiculite Schist seu、Crocus 、Semen Nigellae、 Cultured Calculus Bovis、Artificial musk |
| Jiuwei Niuhuang Wan | Safflower 、Herba corydalis impatiens、elecampaneAristolochia debilis 、Bovis、faeces ochotonae erythrotis、Herpetospermum caudigerum Wall、Swertia bimaculata、Herba gentianae urnulae、elecampane |
| Lishukang Capsule | conic gymnadenia tuber、Dracocephalum tanguticum Maxim、Rhodiola、Rhododendron、Phellodendron、licorice |
| LiuGanWan | Terminalia chebula、rheum officinale 、elecampane、Swertia bimaculata、Tibet inula root、Cremanthodium reniforme、Cloves、Oxytropis falcate Bunge、Embelia rudis、erect hypecoum、 Ferula asafoetida、Bangga、Euphorbia paste、radix aconiti agrestis、benzoin、Tibetan calamus、keel、Artificial musk、Tinospora cordifolia、calculus bovis factitius、cardamom |
| LiuWei Nengxiao Wan（Capsule） | Tibet inula root、Dried ginger、Terminalia chebula、Rhubarb、 gypsum rubrum 、Alkali flowers |
| Nuodikang Pill（Capsule、Granule、Oral solution） | Rhodiola |
| Pazhu Wan(Capsule） | gypsum rubrum (processed with wine)、cinnamon、Pomegranate seeds、pepper 、Dried ginger、Safflower、Piper longum、Terminalia chebula（denucleate）、cardamom、Bright eyes、elecampane |
| Qiwei Honghuashusheng Powder（Pill） | Safflower、Tabasheer、Swertia bimaculata、Terminalia chebula、Ephdra vulgaris 、elecampaneAristolochia debilis、Herba gentianae urnulae |
| Ruyi Zhenbao Wan | Mother of pearl、agilawood、travertine、Vermiculite Schist seu、Safflower、crab、Cloves、Terminalia terminalia（denucleate）、Myristica fragrans Houtt、cardamom、Emblic leafflower fruit 、Amomum 、Vermiculite Schist seu、sandalwood、Semen Nigellae、dalbergia wood、Terminalia chebula、Alpinia officinarum Hance、licorice cream、cinnamon、Frankincense、elecampane、Cassia seeds、Buffalo horn、Ambrette、Lagotis integra、Tibet inula root、Artificial musk、Bovis |
| Thirty five flavor Chenxiang Wan | agilawood、yellow cinnamon root or stem 、white agilawood、sandalwood、dalbergia wood、Tabasheer、Safflower、Cloves、Myristica fragrans Houtt、cardamom、Amomum 、Terminalia chebula(denucleate)、Terminalia terminalia(denucleate)、Emblic leafflower fruit (denucleate)、elecampane、fructus choerospondiatis 、Tibet inula root、Hanging hook wood、Tinospora cordifolia、resurrection lily rhizome、kapok、nuces vomicae、Frankincense、benzoin、Herba corydalis impatiens、rockfoil 、Lagotis integra、Herba gentianae urnulae、Flos Pyrethri Tatsienensis、Cremanthodium reniforme、seed radish、Pomegranate seeds、Iron hammer、Bison heart、Artificial musk |
| ShanHuQiShiWei Wan | Coralline、pearl、carnelian 、Angelica sinensis、Tibetan party ginseng、Rhodiola、lanatechead saussurea herb with flower 、Emblic leafflower fruit 、Tibetan Safflower、rhizoma polygonati、natural bovis、Artificial musk etc. |
| Shiwei Dida Capsule | Tida、Herba Lagotis 、Bangga、Herpetospermum caudigerum Wall、erect hypecoum、Ixeris chinensis nakai、Chrysosplenium nudicaule Bge、cortex berberidis、elecampane、Bear bile powder |
| Shiweiheibingpian Wan | Black ice sheet、Pomegranate seeds、cinnamon、cardamom、Piper longum、Terminalia chebula、natural salt、Herpetospermum caudigerum Wall、Stop diarrhea rice、bear gall |
| Shiwei Longdanhua Granule（Capsule） | Gentian、Rhododendron、Fritillaria roylei Hooker、licorice、Short purple pansy、 cuticle 、Tibet inula root、younghusband jerusalemsage root、convolvulate asiabell root 、radix przewalskiae tanguticae |
| Shiwuwei Heiyao Wan | gypsum rubrum 、Salt (fried)、Rhododendron、Tibetan akebia quinata Decne、Myristica fragrans Houtt、Coriander、Glauber salt、sal ammoniac、natural salt 、sal ammoniac、Bangga、Tibet inula root、Piper longum、black pepper、Dried ginger |
| ShiWuWeiLongDanHua Wan | Gentian、sandalwood、Terminalia chebula、Terminalia terminalia、Emblic leafflower fruit、travertine 、elecampane、fructus choerospondiatis、Cloves、Myristica fragrans Houtt、Tinospora cordifolia、agilawood、Herba corydalis impatiens、acaulescent pegaeophyton herb、licorice |
| Pomegranate Jianwei Wan(Pill、Capsule、Powder） | Pomegranate seeds、cinnamon、Piper Ba、Safflower、cardamom |
| ZhituoJiebai Wan | gypsum rubrum 、Short purple pansy、Terminalia chebula、Lagotis integra、elecampane、 honey、faeces ochotonae erythrotis |
| Zuozhu Daxi | gypsum rubrum 、travertine、Tabasheer、Aconitum napellus、Crocus 、Myristica fragrans Houtt、Amomum 。Crocus 、bear gall、Bovis、musk etc. |
| Anshen Wan | Areca catechu、agilawood、Cloves、Myristica fragrans Houtt、elecampane、fructus choerospondiatis 、 resurrection lily rhizome、Piper longum、 black pepper、sal ammoniac、Iron hammer、Rabbit heart 、Bison heart、 Ferula asafoetida、brown sugar |
| Liuwei Mingmu Wan | Iron powder、cortex berberidis、caraway、Terminalia chebula、Terminalia terminalia、Emblic leafflower fruit |
| Shiliuwei Dongqing Wan | folia ilicis 、pomegranate、oulopholite、cinnamon、cardamom、elecampane、Cloves、licorice、White raisins、agilawood、polygonum bistorta，Piper longum、Myristica fragrans Houtt、Safflower、fructus choerospondiatis 、Fanghai |
| Qiwei Guangzao Wan | fructus choerospondiatis 、Myristica fragrans Houtt、Cloves、elecampane、resin of sweetgum 、agilawood、Cow heart powder |
| Qiweiputao Powder | White raisins、oulopholite、Safflower、licorice、rhizoma cyperi 、cinnamon、pomegranate |
| Qingxinchenxiang Bawei Powder/Bawei Qingxinchenxiang Powder | agilawood、fructus choerospondiatis 、sandalwood、sandalwood、Safflower、Myristica fragrans Houtt、Tabasheer、radix glehniae |
| Bawei Tanxiang Powder | sandalwood、oulopholite、Safflower、licorice、Cloves、oulopholite、Polygonum amplexicaule、White raisins |
| Sanzisan | Terminalia chebula、toosendan fruit、Gardenia |
| Sanwei tribulus Powder | Tribulus teristus、Fructus Malvae Vertillatae、Fanghai |
| Wuwei Shaji Powder | Seabuckthorn cream、Gardenia、elecampane、licorice、White raisins |
| WuweiQingzhuo Powder | pomegranate、Safflower、cardamom、cinnamon、Piper longum |
| Liuwei Muxiang Powder | elecampane、Gardenia、pomegranate、 Rhododendron molle G.Don、cardamom、Piper longum |
| Siwei Tumuxiang Powder | elecampane、 kuh-seng、 Hanging hook wood（remove the peel and heart）、 resurrection lily rhizome |
| Alatan Wuwei Wan | Terminalia chebula、pomegranate、 Momordica cochinchinensis、excrementum pteropi、Black ice sheet |
| Anshen Buxin Liuwei Wan | bovine heart、elecampane、 resin of sweetgum 、Cloves、Myristica fragrans Houtt、fructus choerospondiatis |
| Bateri Qiwei Wan | radix aconiti agrestis leaf、Terminalia chebula、potentilla discolor、Rubia cordifolia、 Resin Commiphorae muakulis、Artificial musk、cinnabar |
| Dhuang Sanwei Pill | Rhubarb、Terminalia chebula、baking soda |
| FengShi Ershiwuwei Wan | Donkey blood powder、sandalwood、sandalwood、 kuh-seng、Gardenia、 Rhododendron molle 、calculus bovis factitius、Crocus 、Amomum 、cardamom、tokyo violet herb、Terminalia chebula、toosendan fruit、Artificial musk、Leak reed flowers、oulopholite、flower of fragrant plantain lily 、Myristica fragrans Houtt、 chingma abutilon seed、 resin of sweetgum 、 Cassia occidentalis、kapok、Cloves、tu-chung |
| Hanshuishi Ershiyiwei Powder | gypsum rubrum 、pomegranate、 sea-buckthorn 、excrementum pteropi、fructus amomi 、Piper longum、tokyo violet herb、 Momordica cochinchinensis、calculus bovis factitius、Forsythia suspensa Vahl、moldavica dragonhead 、elecampane、Coriander、Blue basin flower、 fringed pink、Acid pear stem、elecampane、dalbergia wood、 Radix Ophiopogonis、Terminalia chebula、Gardenia |
| Honghuaqinggan Shisanwei Wan | Safflower、Cloves、semen nelumbinis 、 Radix Ophiopogonis、elecampane、Terminalia chebula、toosendan fruit、Gardenia、sandalwood、Artificial musk、Buffalo horn、calculus bovis factitius、cinnabar |
| HuangBaiBaWei Pill | Phellodendron、Fragrant ink、Gardenia、licorice、Safflower、Piper longum、bilein、 Resin Commiphorae muakulis |
| Jixiang Ankun Wan | leenurus heterophyllus 、 sea-buckthorn 、Red eggplant son、Terminalia chebula、excrementum pteropi、Safflower、elecampane、 resurrection lily rhizome、Burnt leaves、elecampane、cartialgenous、Small white artemisia、Cloves、Cinnabar、calculus bovis factitius、Cordyceps sinensis、bilein 、 borax |
| Naru Sanwei Wan | Terminalia chebula、 Piper longum、radix aconiti agrestis |
| Nuangong Qiwei Wan（Powder） | cardamom、Asparagus cochinchinensis、conic gymnadenia tuber、agilawood、Myristica fragrans Houtt、rhizoma polygonati、Cloves |
| Qinggan Jiuwei Wan | radix aconiti agrestis、Terminalia chebula、elecampane、 Resin Commiphorae muakulis、Leak reed flowers、Rhizoma Picrorhizae、Polygonum amplexicaule、oulopholite、potentilla discolor |
| Qingre Bawei Wan（Powder、Capsule） | sandalwood、oulopholite、Safflower、bunge corydalis herb 、 fringed pink、Rhizoma Picrorhizae、 Radix Ophiopogonis、calculus bovis factitius |
| Roukou Wuwei Wan | Myristica fragrans Houtt、elecampane、elecampane、fructus choerospondiatis 、Piper longum |
| Surilao Qingfei Zhike Capsule | oulopholite、Terminalia chebula、toosendan fruit、Gardenia、lacca、 lithospermum、Rubia cordifolia |
| Diaoyuandabu Ershiwuwei Tangsan | Safflower、Terminalia chebula、toosendan fruit、Gardenia、elecampane、elecampane、bunge corydalis herb 、Rhizoma Picrorhizae、Radix Gentianae Macrophyllae、 Radix Ophiopogonis、pomegranate、Acid pear stem、Cyrtomium fortunei 、Gentiana siphonantha、flos chrysanthemi indici 、Asarum sieboldi Mig.、Coriander fruit、Momordica cochinchinensis、Pig blood powder、flos farfarae、Blue basin flower、 fringed pink、moldavica dragonhead 、excrementum pteropi、cardamom |
| Wulan Shisanwei Tangsan | 土elecampane、 kuh-seng、 Hanging hook wood、 resurrection lily rhizome、Terminalia chebula、toosendan fruit、Gardenia、Rubia cordifolia、folia eriobotryae 、lacca 、 acorn 、lithospermum、 trollflower |
| Xiaoji Jiebai Wan | Ten thousand years of ash、 resurrection lily rhizome、sal ammoniac、 sea-buckthorn 、Piper longum |
| Yishen Shiqiwei Wan | Terminalia chebula、radix aconiti agrestis、Acorus gramineus Soland、elecampane、 the shell of abalone or sea-ear(calcine)、cinnabar、bilein 、Resin Commiphorae muakulis、Canavalia gladiata、Rubia cordifolia、Safflower、folia eriobotryae 、Fragrant ink、Artificial musk、cardamom、Big shu season flower、lacca |
| Xiaoer Shikou Powder | pomegranate、cynomorium songaricum 、swampy gentianopsis herb 、Safflower、keel(charcoal)、Garlic(charcoal)、Black ice sheet(charcoal)、cinnamon、Piper longum、excrementum pteropi(charcoal)、cardamom、Alpinia oxyphylla 、 gypsum rubrum |
| Zhachong ShisanweiWan | Terminalia chebula、radix aconiti agrestis、Acorus gramineus Soland、elecampane、Artificial musk、Coralline、pearl、Cloves、Myristica fragrans Houtt、agilawood、red claystone、magnetite(calcine)、licorice |
| Zhenbao Wan | oulopholite、Cloves、Terminalia chebula、toosendan fruit、Gardenia、Safflower、Myristica fragrans Houtt、cardamom、Cassia seeds、Amomum 、Annel、 resin of sweetgum 、elecampane、elecampane、licorice、sandalwood、dalbergia wood、humifuse euphorbia herb、Bai Jusheng、Semen Nigellae、Fanghai、Lygodium japonicum、agilawood、Piper longum、cinnamon、Artificial musk、calculus bovis factitius、pearl、Buffalo horn |
| Zhenzhu tongluo Wan | pearl、oulopholite、Safflower、Cloves、Myristica fragrans Houtt、cardamom、Amomum 、calculus bovis factitius、sandalwood、agilawood、humifuse euphorbia herb、Fanghai、Artificial musk、elecampane、Piper longum、cinnamon、Terminalia chebula、toosendan fruit、Gardenia、Lygodium japonicum、Fructus Malvae Vertillatae、White chicory wins、Black chicory wins、Buffalo horn |
| Liangxue Shiwei Powder（Pill） | lithospermum、 gypsum rubrum 、elecampane、Rhizoma Picrorhizae、 fringed pink、oulopholite、Safflower、licorice、elecampane、oulopholite |
| Aiweixin oral solution | silkworm cocoon、Anchusa azurea、rhizoma nardostachyos、Yellow flower willow flower、Artificial musk、Crocus 、moldavica dragonhead 、 lavender 、Anchusa azurea flower、cardamom、Song luo、Flos rosae rugosae 、Myristica fragrans Houtt、Cloves，Tibet cinnamon bark |
| Baixuanxiatare Pill（Capsule） | humifuse euphorbia herb、Terminalia chebula、Terminalia terminalia、  Scarmonia fat、barbados aloe、ructus terminaliae immaturus |
| Compound gaoziban pills | Anchusa azurea、Cornflower root、sandalwood、Herba macrophylla、moldavica dragonhead 、Semen Lepidii sativi、perillae、Anchusa azurea flower、 silkworm cocoon、lavender 、Coriander fruit |
| Compound Muniziqi Granule | seed of Cichorium inrybus、Celery root、Chicory root、moldavica dragonhead fruit、Semen Nigellae、the peel of fennel root、Chamomile、licorice、 citronella、Basil seeds、Hollyhock seed、Anise fruit、The camel salicornia |
| Hanchuan Zupa Granule | Vanilla、capillaire 、licorice extractum 、fennel、celery seeds、 fenugreek、Remote Lemongrass Herb、roses、The seed of the nettle |
| Zukamu Granule | rhizoma kaempferiae 、Nymphaea candida Presl、Broken wood fruit、Peppermint、Jujube、Chamomile、licorice、Hollyhock seed、Rhubarb、pericarpium papaveris |
| Hugan Buzure Granule | celery seeds、seed of Cichorium inrybus、Cuscuta、Celery root 、the peel of fennel root、Chicory root、fennel |
| Jianxin hemilgaozi ban'an pirated tablets | Anchusa azurea、Anchusa azurea flower、 ambergris 、pearl、amber、 silkworm cocoon、Coralline、sandalwood、gold foil、silver paper、ruby、Yellow flower willow flower、moldavica dragonhead 、roses |
| Luobufukebiri Wan | bullwhip、cinnamon、Alfalfa、Alpinia officinarum Hance、Onion seed、Cloves、turnip seed 、Chinese prickly ash、carrot seed 、Sesamum indicum、semen melo、Amygdalus communis 、Yellow melon seeds、Asarum europaeum 、semen allii tuberosi 、Herba macrophylla、dill、Tribulus teristus、Milk peach、common four-o'clock root 、cotton seed 、elecampane、ginger flake、Myristica fragrans Houtt、 nagkassar 、long pepper、Myristica fragrans Houtt、White skin pine son、Sesamum indicum seed、Crocus |
| Mamulan antidiarrheal capsule | the rhizome of Chinese goldthread、dragon's blood、Frankincense、nutgall、gum trargacanth、pomegranate flower、Tabasheer、plantago seed、ructus terminaliae immaturus、Coriander、Terminalia chebula、Small berry fruit etc. |
| Meiguihua oral solution | Fresh roses |
| Niaotongkakenaiqi Pill | Yellow melon seeds、dragon's blood、 gum trargacanth、wintercherry、gummi arabicum 、Bardenren、licorice extractum 、Frankincense、celery seeds、 gum opium |
| Qingre Carson Granule | witloof |
| Pomegranate blood tonifying syrup | pomegranate、pomegranate |
| Tongzhisurunjiang Capsule（Pill） | meadow saffron、Scarmonia fat、Crocus 、folium sennae 、Terminalia chebula、Operculina turpethum、Bardenren |
| Yanxiao Dinar syrup | Chicory root、seed of Cichorium inrybus、roses、Rhubarb、Nymphaea candida Presl、Anchusa azurea、Cuscuta |
| YangXinDaWaYiMiXiKeMiGao | musk、sandalwood、sandalwood、pearl、frankincense、cinnamon、Anchusa azurea flower、silkworm cocoon、agilawood、Crocus 、Operculina turpethum、Tabasheer、berberis、Herba macrophylla、gold foil、silver foil、amber、Song luo、Chamomile root、moldavica dragonhead 、apple、Coriander seed、roses、cardamom、purslane seed 、Emblic leafflower fruit |
| Yixin badiranjibuya Granule | moldavica dragonhead |
| YajiaoHadun San | officinal asparagus root、kuh-seng rattan、Bitter wax gourd、Tacca chantrieri、sheepear inula herb、fructus viticis |
| HufengJiu | wasp、wine |
| Duyiwei Capsule(pill) | Herba Lamiophlomis |
| Anerning Granule | Tabasheer、Safflower、calculus bovis factitius、 purple bergenia herb、licorice、Mountain horseradish、Lagotis integra、sandalwood、the rhizome of Chinese monkshood |
| Honghua Ruyi Wan | Safflower、Crocus 、Radix podophylli、Terminalia chebula、Tibetan rubia cordifolia、cinnamon、Herba corydalis impatiens、Tibet inula root、Coriander fruit、dalbergia wood、Bear bile powder、Tibetan lithospermum、natural salt 、Himalayan four-o'clock root 、Bangga、black pepper、Snake meat（detoxicate）、Short purple pansy、Emblic leafflower fruit 、Seabuckthorn cream、sal ammoniac、lacca、the fruit of Chinese wolfberry、agilawood、possium nitrate ta |
| Dengzhan Shengmai Capsule | Erigeron breviscapus 、ginseng、the fruit of Chinese magnoliavine、 Radix Ophiopogonis |
| Yindan Xinnaotong Capsule | ginkgo leaf、the root of red-rooted salvia 、Erigeron breviscapus、Gynostemma pentaphylla 、 hawthorn、garlic、pseudo-ginseng 、blumea camphor |
| Lingdancao Granule | Herba Laggerae,The auxiliary materials are sucrose, lactose and dextrin |

**Table S5**

The Characteristics table of CPEs

| Drug Name | usage | Single dose/day | Use frequency | Market unit price (Yuan) / g (ml) | Market price (Yuan) / day | Components of the medicines | Symptoms and signs that medicines treat | Diseases that medicines treat | Treatment period |
| --- | --- | --- | --- | --- | --- | --- | --- | --- | --- |
|  |  |  |  |  |  |  |  |  |  |
|  |  |  |  |  |  |  |  |  |  |
|  |  |  |  |  |  |  |  |  |  |
|  |  |  |  |  |  |  |  |  |  |

**Table S6**

The Clinical research characteristics table of CPEs

| Author  (year) | Identified medicines and target conditions | Sample size（T/C） | Interventions  （T/C） | Period of treatment | Study design | Outcomes | Reatment adverse events and the types of adverse events |
| --- | --- | --- | --- | --- | --- | --- | --- |
|  |  |  |  |  |  |  |  |

T:Treatment group

C:Control group
